# Extra-foveal Processing of Object Semantics Guides Early Overt Attention During Visual Search

**DOI:** 10.3758/s13414-019-01906-1

**Published:** 2019-12-02

**Authors:** Francesco Cimminella, Sergio Della Sala, Moreno I. Coco

**Affiliations:** 1 Human Cognitive Neuroscience, Psychology, University of Edinburgh, Edinburgh, UK; 2 Laboratory of Experimental Psychology, Suor Orsola Benincasa University, Naples, Italy; 3 School of Psychology, The University of East London, London, UK; 4 Faculdade de Psicologia, Universidade de Lisboa, Lisbon, Portugal

**Keywords:** Visual search, Eye movements, Object semantics, Early overt attention, Extra-foveal vision

## Abstract

Eye-tracking studies using arrays of objects have demonstrated that some high-level processing of object semantics can occur in extra-foveal vision, but its role on the allocation of early overt attention is still unclear. This eye-tracking visual search study contributes novel findings by examining the role of object-to-object semantic relatedness and visual saliency on search responses and eye-movement behaviour across arrays of increasing size (3, 5, 7). Our data show that a critical object was looked at earlier and for longer when it was semantically unrelated than related to the other objects in the display, both when it was the search target (target-present trials) and when it was a target’s semantically related competitor (target-absent trials). Semantic relatedness effects manifested already during the very first fixation after array onset, were consistently found for increasing set sizes, and were independent of low-level visual saliency, which did not play any role. We conclude that object semantics can be extracted early in extra-foveal vision and capture overt attention from the very first fixation. These findings pose a challenge to models of visual attention which assume that overt attention is guided by the visual appearance of stimuli, rather than by their semantics.

**Electronic supplementary material:**

The online version of this article (10.3758/s13414-019-01906-1) contains supplementary material, which is available to authorized users.

When searching for an object in a visual context, such as a photograph, an array of objects or a richer 3D environment, bottom-up stimulus driven information (i.e., low-level), as well as top-down knowledge based information (i.e., high-level), are effortlessly integrated to guide our visual attention to the regions of the context where such object could be more likely found (see J. M. Wolfe & Horowitz, 2004, 2017; Wu, Wick, & Pomplun, 2014 for reviews).

The seminal Feature-Integration Theory (FIT) by Treisman and Gelade (1980) attributes a key role to the low-level, or *visual*, features of stimuli (e.g., colour, shape, orientation) when explaining visual search behaviour, and assumes a two-stage architecture. During the first stage, all visual features are pre-attentively, independently and simultaneously (in parallel) processed across the visual field in a bottom-up fashion. Then, in the second stage, overt attention is serially directed to bind such features into unitary objects. The two stages are not independent: the visual information gathered during the pre-attentive, parallel stage, is used to guide visual attention during the serial stage (Treisman & Sato, 1990). A similar proposal was developed in the Guided Search (GS) model (J. M. Wolfe, Cave, & Franzel, 1989) which assumes that visual features of objects can contribute to visual search both in a bottom-up and top-down fashion (J. M. Wolfe, 1994).

Since then, there has been a proliferation of computational models of attention especially relying on low-level visual features. One of the most prominent is the visual saliency model by Itti and Koch (2000), which is based on a composite measure of low-level visual information (e.g., brightness, contrast, and colour), and can be used to simulate how overt attention may unfold in a given visual context (e.g., Walther & Koch, 2006). Bottom-up saliency models may effectively predict overt attention when the visual search is not cued to any specific target object, and such target differs in visual features, e.g., colour, from other homogeneous distractors (J. M. Wolfe, Butcher, Lee, & Hyle, 2003).

When the identity of the target is instead cued prior to the search, through a word label or a visual object (e.g., Malcolm & Henderson, 2009, 2010), low-level visual features are largely ignored (e.g., Chen & Zelinsky, 2006), and overt attention is mostly guided in a top-down fashion to regions of the visual context that contain high-level knowledge based information related to the search target (Zelinsky, 2008; Zelinsky, Adeli, Peng, & Samaras, 2013). For example, when searching for a red ball, observers will preferentially look at visually similar (e.g., a red apple) than dissimilar (e.g., a yellow banana) objects (e.g., Alexander & Zelinsky, 2011; Schmidt & Zelinsky, 2009). This effect does not relate only to visual information, such as similarity in colour between objects, but it extends to conceptual information, such as their semantic relationships (Wu et al., 2014). In fact, observers tend to prioritise distractors that are semantically related (e.g., an anchor) with a target (e.g., a ship) than unrelated (e.g., a rabbit) with it, especially on target-absent trials, in standalone object arrays (e.g., Belke, Humphreys, Watson, Meyer, & Telling, 2008; de Groot, Huettig, & Olivers, 2016; Moores, Laiti, & Chelazzi, 2003).

Although these studies agree that semantic information can guide the allocation of overt attention, there are some controversies about the time-course of processing. Moores et al. (2003) and Belke et al. (2008) reported semantic relatedness effects on the very first saccadic eye movement after the onset of the object array. However, Daffron and Davis (2016) claimed that this evidence might have been confounded by the repeated exposure of the stimuli to the participants. For example, in Belke et al.(2008), participants inspected the visual stimuli (line drawings of objects) before the experiment began, thus raising the concern that eye movements were guided by the memory of the visual features of the stimuli rather than by their semantics. Instead, de Groot et al. (2016) used each stimulus only once and found that early visual attention was primarily driven by the visual similarity between the objects, whilst semantic information would mainly influence later eye movements (but see Nuthmann, de Groot, Huettig, & Olivers, 2019 for a re-analysis of this data showing much earlier semantic effects).

Evidence of semantic relatedness on early overt attention also directly speaks about the degree of semantic processing that may happen outside the fovea. Conventionally, the visual field is characterised by three regions, going from the centre to the periphery of the retina: (1) the fovea, which subtends a visual angle of 1° eccentricity and is responsible for high resolution vision; (2) the parafovea, which stretches out to 4-5°; and (3) the periphery, which extends beyond the parafovea and cover the rest of the visual field (see Larson & Loschky, 2009 for provising a brief summary in the context of scene gist recognition). Although the visual acuity strongly decreases in the parafovea and in the periphery, i.e., in extra-foveal vision (e.g., Strasburger, Rentschler, & Jüttner, 2011), the area of the visual field from which observers can accrue useful information is quite large (see Rayner, 2014; Rosenholtz, 2016; B. Wolfe, Dobres, Rosenholtz, & Reimer, 2017, for reviews) and it can roughly corresponds to 8° in each direction from fixation for visual search in naturalistic scenes (Nuthmann, 2013). Previous studies have found that object semantics are accessed in extra-foveal vision as early as at the onset of the object array (Auckland, Cave, & Donnelly, 2007; Gordon, 2004), but as the visual stimuli were presented very quickly and eye movements were not recorded, it is still unclear whether object semantics were processed to a degree sufficient to guide overt attention from the very first fixation.

The involvement of extra-foveal semantic processing on the early guidance of overt attention clashes with standard definitions of FIT (Treisman & Gelade, 1980; Treisman & Sato, 1990) and with more recent models of visual search (J. M. Wolfe, 2007; Zelinsky, 2008; Zelinsky et al., 2013), where such guidance would purely depend on the visual features of stimuli. That being said, more recent updates of FIT (Evans & Treisman, 2005; Treisman, 2006) do not rule out the possibility that some semantic features of objects, e.g., category membership, can be detected in the periphery of the visual field, and hence guide overt attention (see also Zelinsky et al., 2013, on page 10, which despite presenting a computational model of categorical search entirely relying on visual features of objects, does not entirely rule out a possible role for semantic features).

The current visual search study aims to shed new light on the time-course of extra-foveal processing of object semantics while providing more conclusive evidence about its impact on the very first eye-movement responses, i.e., the first deployment of overt attention.

In our task, participants were presented with a cue word for a critical object to be searched in an array with 2, 4 or 6 additional semantically homogenous distractor objects (e.g., all *vehicles*). We manipulated the visual saliency of the critical object (salient or non-salient) as well as its semantic relatedness (related or unrelated) with the other distractors (see Figure 1 for the experimental design and materials and refer to the Method section for more details). Each object was located with an eccentricity of 9.62° of visual angle from the centre of the screen, i.e., it was placed in extra-foveal vision. On target-present trials, the cue word referred to the critical object, which was the target of the search. On target-absent trials, the cue word, instead, referred to an object that did not appear in the array, and it was semantically related to the critical object (e.g., the cue word was *airplane* and the critical object was instead a *car*).

**Fig. 1:**
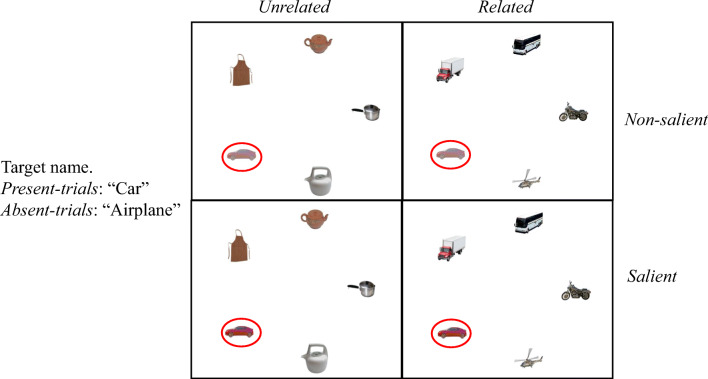
Experimental design and example of an object array, which included a critical object (e.g., **car**, highlighted in red) plus either **2**, **4**, or **6** distractors. On target-present trials, the target name cued the critical object as the target. On target-absent trials, the target name cued an object that was not visually depicted in the array, but it was semantically related to the critical object and thus to the distractors in the semantically related but not the unrelated condition (e.g., airplane)

Our manipulation of semantic relatedness differs from previous studies (De Groot et al., 2016; Nuthmann et al., 2019), and we hypothesize that it is precisely this aspect of the experimental design that may increase the probability to observe effects of semantic guidance on early overt attention. In these studies, a competitor object semantically related to the target was presented together with distractors that were semantically unrelated to the target, to the semantic competitor, and among themselves. For example, participants searched for a target (e.g., a banana) in an object array comprising a semantic competitor (e.g., a monkey), a visually similar competitor (e.g., a canoe) and two more unrelated distractors (e.g., tambourine and hat). A seminal study by Duncan and Humphreys (1989) showed that when distractors differ homogenously from the target on a target-defining visual feature (e.g., the colour), the guidance of such feature on directing overt attention is very strong. When distractors are instead more heterogeneous, then such a feature has a weaker effect. We followed the same logic in our study but applied it to semantic relatedness.

We expected the critical object to capture overt attention earlier when semantically unrelated than related to the distractors, which were all semantically related to each other.

More specifically, if object semantics can be processed early in extra-foveal vision and guide overt attention promptly, we expected to observe semantic relatedness effects on the probability of the very first fixation after the onset of the array, and be corroborated by the measure of search latency, i.e., the time it takes for the critical object to be look at for the first time. In line with previous literature (Belke et al., 2008; Moores et al., 2003), we also expected the presence of the target in the array to reduce the effects of semantic relatedness because participants might rely more on visual information to facilitate search (Huettig & Altmann, 2005; Huettig & Mcqueen, 2007).

We also manipulated the size of the distractor set in our experimental design. We did this to test if, and to what degree, semantic processing can occur in parallel across the visual field. In fact, if the semantics of all objects in the array are computed in parallel, then a critical object that is semantically unrelated to the other distractors should display exactly the same advantage to be prioritized over a semantically related critical object even when increasing the number of the distractors (i.e., a “pop-out effect”).

All predictions above are about the time-course of target identification, but important differences of object-object semantic integration may also manifest in the processing time, such as in the duration of the first fixation to the critical object. For example, in a visual memory task, Henderson, Pollatsek, and Rayner (1987) found shorter first fixation durations on a critical object when it was presented together with semantically related than unrelated distractors. They explained this result in terms of positive priming arising from having previously fixated semantically related objects. In line with this result, we expected to extend this finding to a visual search task, and hence find shorter first-fixation durations to the critical object when semantically related as opposed to unrelated to the distractors.

Finally, the manipulation of the visual saliency of the critical object allowed us to examine the influence of low-level visual information on overt attention and exclude that it may play a role in a cued visual search, as previously shown by Chen and Zelinsky (2006).

## Methods

### Participants

A total of 144 participants (103 female), students at the University of Edinburgh and aged between 18 and 30 years (*M* = 20.88, *SD* = 2.91), participated in the study for either course credits or a £3.50 honorarium. All participants were native English speakers and had normal or corrected-to-normal vision. Participants were naive to the purpose of the study and unfamiliar with the stimulus material. The study was approved by the Psychology Research Ethics Committee (Ref: 12-1617) prior to starting the data collection, and written consent was collected at the beginning of each session.

### Design

A 2x2x2x3 mixed factorial design was used with two within-participant variables, *Semantic Relatedness* (unrelated, related) and *Visual Saliency* (non-salient, salient) and two between-participant variables, *Target* (absent, present)^1^ and *Set Size* (3, 5, 7)^2^ (see Figure 1). We had 24 participants for each of the six conditions obtained by crossing the between-participants variables of target and set size.

### Stimuli

The visual contexts used for the search task were arrays of either three, five or seven pictures of real-world objects obtained from the Bank of Standardized Stimuli (BOSS) database (Brodeur, Dionne-Dostie, Montreuil, & Lepage, 2010; Brodeur, Guérard, & Bouras, 2014). They were placed on a uniform white background, presented at a resolution of 1024 x 768 pixels, at a viewing distance of 82 cm (28.07° and 21.40° of visual angle on the horizontal and on the vertical axis, respectively). Object pictures had a size of 150 x 150 pixels (4.18° x 4.23° of visual angle) and were arranged on an imaginary circle such that the midpoint of each object was equidistant from the centre of the array (corresponding to the starting fixation point) and from the two adjacent object midpoints. The circle had a fixed radius of 344 pixels (9.62°) while the distance between objects changed depending on the number of objects in the array: 595.83 (16.67°), 404.40 (11.31°), and 298.51 (8.35°) pixels for set size 3, 5 and 7, respectively.

A set of 224 object pictures were used to create the experimental arrays. All the objects were accurately nameable and univocally classifiable into 20 semantic categories (the reader is referred to Supplemental Material A for the norming of the materials). We selected 32 objects from the picture set to be used as critical objects^3^. A total of 384 unique experimental arrays were constructed by crossing the visual saliency (non-salient, salient) and the semantic relatedness (unrelated, related) of the 32 critical objects (128 items, i.e., 32 *4), independently for three set sizes (384 items, i.e., 128 * 3); see Supplemental Material B for miniatures of the experimental arrays. The position of the critical object was counterbalanced by rotating it in different locations of the array, in order to account for potential directional biases. Within each set size, no object was presented more than once to avoid any uncontrolled effect that may derive from repeated exposures to the same stimulus.

The visual saliency of the critical object was manipulated by changing its brightness/contrast and hue/saturation with GIMP (Version 2.8.2) and validated using the Walther and Koch's Matlab Saliency Toolbox (2006). We made sure that the critical object was always ranked among the most and the least salient regions of the array in the salient and non-salient condition, respectively. A Wilcoxon signed-rank test confirmed that the critical object was visually more conspicuous in the salient (*Mdn* = 1) than in the non-salient condition (*Mdn* = 4), *p*< .001, *r* = -.62.

The semantic relatedness manipulation was implemented by constructing object arrays with all objects belonging to the same semantic category (related), or, all distractors of the same semantic category but the critical object of a different one (unrelated). We validated the semantic manipulation using Latent Semantic Analysis (LSA, Landauer & Dumais, 1997; Landauer, Foltz, & Laham, 1998), which is a distributional statistical model trained on co-occurrences of words in a text, and has been already used in the context of visual search (e.g., Hwang, Wang, & Pomplun, 2011). For the current study, we used the LSA trained on co-occurrences of words implemented by Hoffman, Lambon Ralph, and Rogers (2013), on labels of objects as normed by Brodeur et al. (2010, 2014). LSA returns a score that indicates the strength of semantic similarity between pairs of objects (between 0 and 1). For each experimental array, we computed the mean semantic similarity score of the critical object with every other distractor. A t-test confirmed that the semantic similarity between the critical object and all other distractors was significantly higher in the semantically related (*M* = .51, *SD* = .23) than unrelated condition (*M* = .01, *SD* = .09), *t*(95) = 19.80, *p*< .001, *r* = .90.

For each set size, we also constructed 32 filler arrays (96 items in total, i.e., 32 * 3) using objects from the BOSS database (Brodeur et al., 2010, 2014) that did not appear in the experimental arrays. Each participant saw the same 32 fillers and 32 unique experimental arrays, which were counterbalanced across the conditions of visual saliency and semantic relatedness using a Latin square rotation, in one specific set size (i.e., either 3, 5, or 7). In both experimental and filler trials, a cue word of the search target, i.e., the target name, was presented at the centre of the screen prior to the onset of the search array. In the target-present condition, the cue word always referred to a critical object depicted in the experimental array, and it did not refer to any object in the filler array (i.e., it was an absent trial). In the target-absent condition, it was the exact opposite. The filler arrays always had an object depicted in it that the cue word referred to, whereas no object was referred by the cue word in the experimental arrays. In order to implement the semantic relatedness manipulation in these experimental trials, we used a cue word that was either semantically related to the critical object in the display but unrelated to all other semantically homogenous distractor objects (*M* = .02, *SD* = .11), or semantically related to all objects (*M* = .31, *SD* = .22), *t*(95) = 11.18, *p*< .001, *r* = .75 (the reader is referred to Supplemental Material C for the list of target-present and target-absent experimental trials). Regardless of whether the target was present or absent in the experimental arrays, we used the filler arrays to guarantee a balanced distribution of yes/no response, as the target of search was visually present on 50% of the total 64 trials performed, and to keep participants actively engaged in the task.

### Visual similarity

Visual objects belonging to the same semantic category are likely to share visual features (e.g., colour, shape), and this may make the critical object visually more similar to the distractors when semantically related than when unrelated (Hwang et al., 2011). In order to examine this scenario, we used the Bank of Local Analyzer Responses (BOLAR) method (Zelinsky, 2003), which provides an aggregate score of visual similarity (from 0 to 1) between pairs of objects on differences measured along their visual feature dimensions (colour, orientation and size); see also Ko, Duda, Hussey, and Ally (2013) and Ko, Duda, Hussey, Mason, and Ally (2014), for examples of similar research using the same method. A

t-test showed that the critical object was visually more similar to semantically related (*M* = .52, *SD* = .14) than unrelated distractors (*M* = .49, *SD* = .14), *t*(95) = 3.36, *p* = .001, *r* = .33. To control for the effects of visual similarity on search, we included it as a quasi-experimental predictor in our models (See the Analyses section for details).

### Apparatus

Visual stimuli were displayed on a 21-inch ViewSonic G225f - CRT monitor with a refresh rate of 60 Hz using an Asus GeForce GT730 graphics card. Eye movements were monitored using an EyeLink 1000 (SR Research) at a sampling rate of 1000 Hz and a spatial resolution of 0.01° of visual angle. Although viewing was binocular, only the dominant eye was tracked (assessed through parallax test). A forehead and chin rest were used to keep participants’

viewing position stable. Stimulus presentation and data acquisition were implemented on Experiment Builder (SR Research, Version 1.10.1630).

### Procedure

**Fig. 2:**
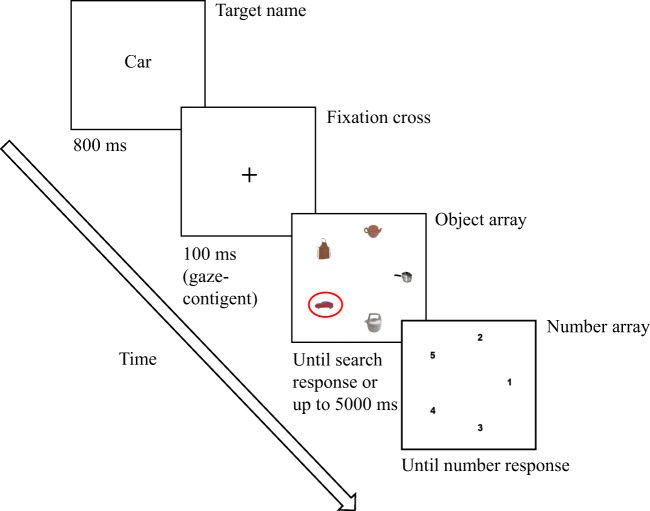
Example of a trial run. The target name was cued at the beginning of the trial. Then, a fixation cross appeared which needed to be fixated for 100 ms to trigger the presentation of the object array. When the participant responded that a target was found, the object array was replaced with a number array. The participant had to indicate then the remembered location of the object in the number array. When the participant responded that the target was not found, the object array was immediately followed by the next trial.

At the beginning of each experimental session, a 9-point calibration and validation procedure were run to setup the eye-tracking accuracy. Each trial began with a drift correction after which a cue word^4^ of the search target was prompted at the centre of the screen for 800 milliseconds (ms), followed by a central fixation cross^5^ and then the object array. Participants received written instruction and asked to indicate, as quickly and accurately as possible, whether the target was present or absent in the object array by pressing the left or the right arrow key on a computer keyboard (search response), respectively. If participants pressed the left arrow key (i.e., the target was present), the object array was

replaced by a number array. Participants were then asked to type in the number matching the target location using the numeric keypad (number response). This provided us with an

additional verification of the search accuracy^6^. If participants pressed the right arrow key (i.e., the target was absent), they moved directly to the next trial. They were given 5000 ms to complete the search, otherwise a null response was logged (see Figure 2 for an example of a trial run). Each participant completed 4 practice trials and 64 randomized trials of which 32

were experimental and 32 filler trials. The experimental session lasted approximately 20 minutes.

## Analyses

### Data pre-processing and exclusion

Raw gaze data were parsed into fixations and saccades using SR Research Data Viewer using the standard setting (i.e., velocity and acceleration thresholds of 30°/s and 9,500°/s^2^, respectively). We annotated each experimental array by drawing bounding boxes around each visual object (i.e., the critical object and all other distractor objects) using LabelMe (Russell, Torralba, Murphy, & Freeman, 2008); see Supplemental Material B to visualize all experimental arrays with the critical object surrounded by the bounding box. Then, we assigned all fixation coordinates to such area of interests. We considered the 4,608 experimental trials only (i.e., 32 trials x 144 participants). Of these trials, we discarded 195 trials because of machine error (no eye movement was recorded). On the remaining trials (4,413), we analysed the response accuracy. The response time was computed on accurate trials only (4,200), whereas eye-movement measures were computed only on accurate trials in which the critical object was fixated at least once (3,967).

### Dependent variables

The performance measures considered in this study are the *response accuracy* (a binary variable coded as 0 = “Incorrect”; 1 = “Correct”), and *response time*, which is the time taken by the participants to provide a yes/no target identification response after the onset of the object array. The response times were log-transformed (natural log-scale) to reduce the positive skew of their distribution. On the eye-movement responses, we computed: (a) the *probability of immediate fixation*, which is a binomial variable indicating whether the first fixation after the onset of the object array (excluding the initial fixation to the centre of the screen) landed on the critical object (0 = “No”; 1 = “Yes”), (b) the *search latency,* which is the time between the onset of the array and the first fixation to the critical object, and (c) the *first-gaze duration*, which is the sum of all consecutive fixations the critical object received for the first time before fixating elsewhere. The probability of immediate fixation as well as the search latency reflect the strength of an object to attract overt attention from the extra-foveal region of the visual field. The first-gaze duration instead is a measure of foveal processing and reflects the difficulty of processing an object once attended.

### Statistical analysis

We used linear and generalized linear mixed-effects models (G/LMM), as implemented by the lme4 package (Bates, Machler, Bolker, & Walker, 2015) in R (version 3.2.5), to analyse the data. In particular, the fixed effects considered, and centred to reduce co-linearity, were: *Semantic Relatedness* (Unrelated = -.5, Related = .5), *Visual Saliency* (Non-salient = -.5, Salient = .5), *Target* (Absent = -.5, Present = .5), *Set Size* (3, 5, 7), where we used the set size of 3 as the reference level, and *Visual Similarity*, which was obtained by splitting the 384 items into two groups (Dissimilar = -.5, Similar = .5) based on the median score obtained with the BOLAR (i.e., 0.502). The random variables included in the models, both as intercepts and slopes, were Participant (144) and Item (384). The model selection procedure is detailed in Appendix A.

## Results

The tables of results report the coefficients, standard errors, t-values (LMM), and z-values (GLMM) of those predictors that were retained in the final models. We also report their p-values based on Satterthwaite approximation for denominator degrees of freedom computed using the lmerTest R package (Kuznetsova, Brockhoff, & Christensen, 2017), whereas p-values in GLMM are based on asymptotic Wald tests. Predictors that were not retained during model selection, because they did not significantly improve the model fit, are not listed in the tables, nor they are plotted in the figures. Moreover, it is worth highlighting that low-level visual saliency was never included, as a significant main effect, in any best-fitting model on any of the measures analysed in this study.

### Accuracy and response time

Response accuracy was at ceiling (target-present: *M* = .93, *SD* = .26; target-absent: *M* = .98, *SD* = .15; *β* = - 4.96, *SE* = .81, *z* = - 6.14, *p*< .001), and hence not further discussed.

**Fig. 3:**
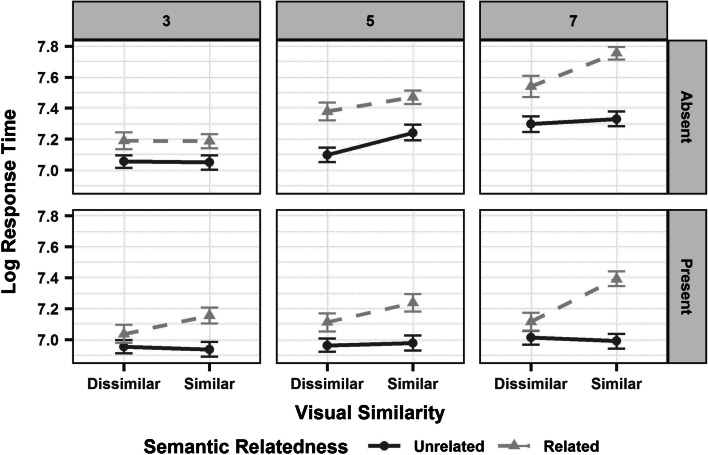
Mean response time (natural-log scale) for set size 3 (Left Panel), 5 (Central Panel) and 7 (Right Panel) on target-present and -absent trials, arranged over the rows of the panels, with the two levels of visual similarity (dissimilar, similar) on the x-axis. The semantic relatedness of the critical object is marked using line types and colour (unrelated: dark grey, solid line; related: light grey, dashed line). Error bars represent 95% confidence intervals around the mean

On response times (Figure 3), we found significant main effects of set size and semantic relatedness. Search responses were faster for set size 3 as compared to 5 and 7, and when the critical object and distractors were semantically unrelated than related. We also observed significant two-way interactions between target and set size, with faster response times on target-present than -absent trials, especially for set size 7; between semantic relatedness and set size, whereby response times were faster when the critical object was semantically unrelated to the distractors, especially for set size 5 and 7; and between visual similarity and set size, whereby response times were faster when the critical object and distractors were visually dissimilar, especially for set size 7. There was also a significant three-way interaction between semantic relatedness, visual similarity and set size, with faster response times when the critical object was semantically unrelated and visually dissimilar to the distractors, especially for set size 7 (See Table 1 for the model output).

**Table 1:** Linear mixed-effects model output for log response time

Dependent Variable	Predictor	β	SE	t-value	Pr ( > | t | )
Log Response Time	Intercept	7.07	0.03	252.93	**< 0.001**
	Semantic Relatedness	0.14	0.03	5.21	**< 0.001**
	Target	- 0.09	0.05	- 1.85	0.07
	Visual Similarity	0.02	0.03	0.77	0.44
	Set size (3 vs. 5)	0.12	0.04	2.98	**0.003**
	Set size (3 vs. 7)	0.24	0.04	6.01	**< 0.001**
	Semantic Relatedness:Visual Similarity	0.08	0.06	1.43	0.15
	Visual Similarity:Set size (3 vs. 5)	0.07	0.04	1.77	0.08
	Visual Similarity:Set size (3 vs. 7)	0.10	0.04	2.42	**0.02**
	Semantic Relatedness:Set size (3 vs. 5)	0.10	0.04	2.44	**0.02**
	Semantic Relatedness:Set size (3 vs. 7)	0.15	0.04	3.80	**< 0.001**
	Target:Set size (3 vs. 5)	- 0.12	0.07	- 1.65	0.10
	Target:Set size (3 vs. 7)	- 0.25	0.07	- 3.49	**< 0.001**
	Semantic Relatedness:Visual Similarity:Set size (3 vs. 5)	- 0.01	0.08	- 0.16	0.88
	Semantic Relatedness:Visual Similarity:Set size (3 vs. 7)	0.20	0.08	2.54	**0.01**
	Semantic Relatedness:Target:Set size (3 vs. 5)	- 0.07	0.05	- 1.50	0.14
	Semantic Relatedness:Target:Set size (3 vs. 7)	- 0.09	0.05	- 1.94	0.05

*Note*. Predictors are listed in the table in the same order as they were entered in the model. The predictors were: target (absent = -.5, present = .5), semantic relatedness (unrelated = -.5, related = .5), visual similarity (dissimilar = -.5, similar = .5), and set size (3, 5, 7). Two planned comparisons were set for set size: 3 vs. 5 (3 = -.5, 5 = .5) and 3 vs. 7 (3 = -.5, 7 = .5).

### Probability of immediate fixation, search latency, and first-gaze duration

**Fig. 4:**
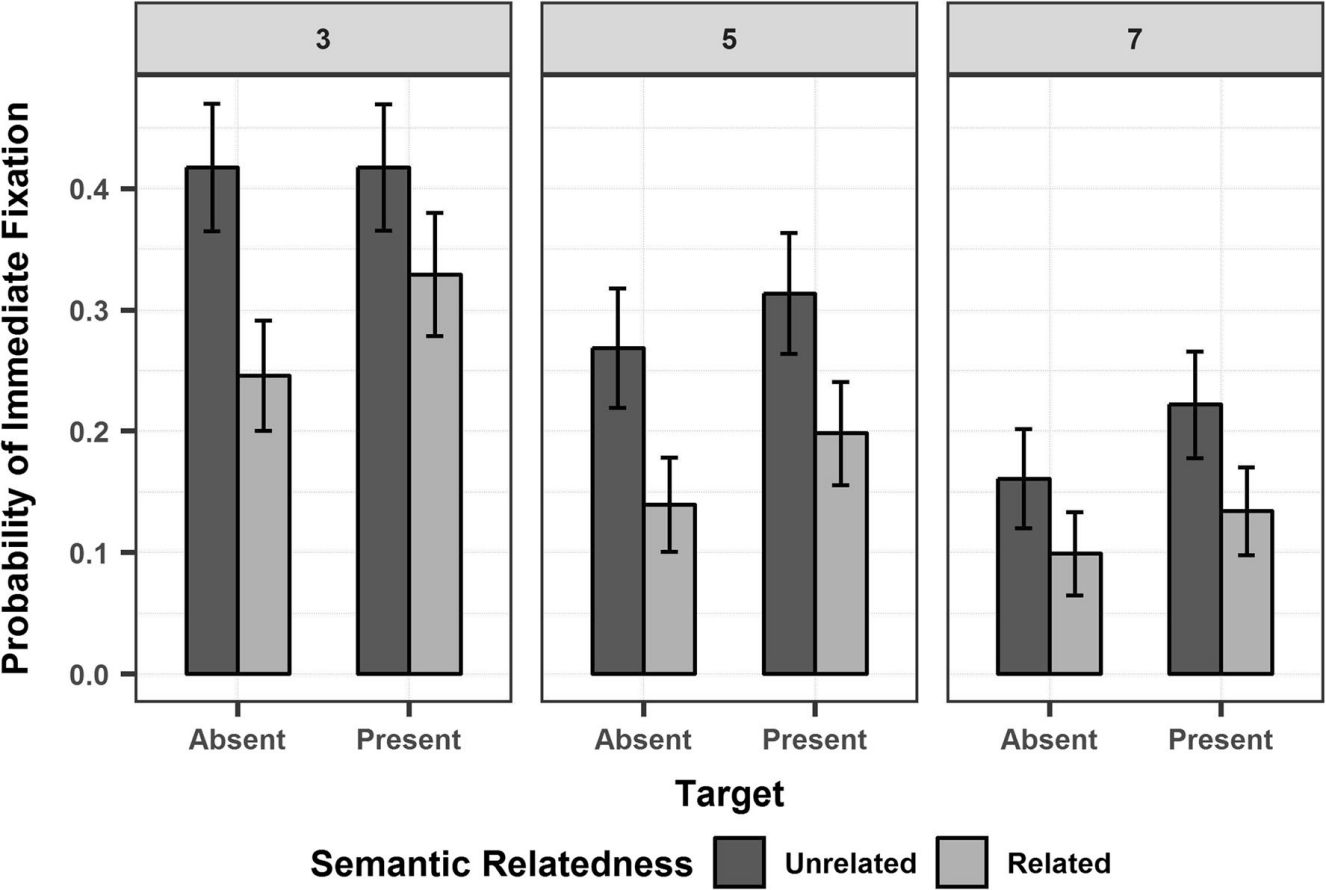
Mean probability of immediate fixation to the critical object (proportions) for set size 3 (Left Panel), 5 (Central Panel), and 7 (Right Panel) on target-present and -absent trials (on the x-axis), in the semantically unrelated (dark grey) vs. related (light grey) condition. Error bars represent 95% confidence intervals around the mean

On the probability of immediate fixation (Figure 4), we found significant main effects of set size, target, and semantic relatedness. The probability of looking at the critical object on the first fixation after array onset was higher for set size 3 than 5 and 7, on target-present than target-absent trials, and when it was semantically unrelated than related to the distractors (Refer to Table 2 for the model output). We also compared the probability of immediate fixation to the critical object, which we call observed probability (OP), for both the semantically related (OP*r*) and unrelated condition (OP*u*) of each set size, with the chance probability (CP) of looking at any object in the array calculated as *1/(N+1)*, where *N+1* is the total number of objects in the array, *N*, plus the blank section of the display (as fixations may also fall outside of the objects). This means that the CP equalled .25, .17, and .13 for set size

**Table 2:** Generalized linear mixed-effects model output for probability of immediate fixation to the critical object

Dependent Variable	Predictor	β	SE	z-value	Pr ( > | z | )
Probability of Immediate Fixation	Intercept Set size (3 vs. 5) Set size (3 vs. 7) Semantic Relatedness Target	- 0.73 - 0.71 - 1.24 - 0.70 0.30	0.10 0.15 0.15 0.12 0.09	- 7.18 - 4.77 - 8.02 - 6.01 3.22	**< 0.001** **< 0.001** **< 0.001** **< 0.001** **0.001**

*Note*. Predictors are listed in the table in the same order as they were entered in the model. The predictors were: target (absent = -.5, present = .5), semantic relatedness (unrelated = -.5, related = .5), and set size (3, 5, 7). Two planned comparisons were set for set size: 3 vs. 5 (3 = -.5, 5 = .5) and 3 vs. 7 (3 = -.5, 7 = .5).

3, 5, and 7, respectively. Under binomial testing, we saw that the OP*u* was significantly higher than CP for set size 3 (*M* = .42, *SD* = .49), 5 (*M* = .29, *SD* = .45), and 7 (*M* = .19, *SD* = .39)(all *ps*<.001), whereas OP*r* did not differ significantly from CP, across all set sizes (3: *M* = .28, *SD* = .45; 5: *M* = .17, *SD* = .38; 7: *M* = .12, *SD* = .32; all *ps*>.05). We obtained identical results for the subset of trials where the very first fixation landed on an object (i.e.,

2872 trials), and the CP was equal to *1/N*, which is .33, .20, and .14 for set size 3, 5, and 7, respectively^7^.

**Fig. 5:**
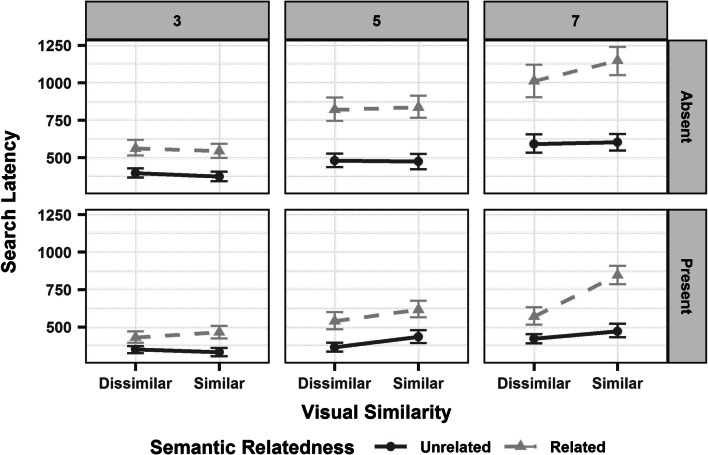
Mean search latency (ms) on the critical object for set size 3 (Left Panel), 5 (Central Panel) and 7 (Right Panel) on target-present and -absent trials, arranged over the rows of the panels, with the two levels of visual similarity (dissimilar, similar) on the x-axis. The semantic relatedness of the critical object is marked using line types and colour (unrelated: dark grey, solid line; related: light grey, dashed line). Error bars represent 95% confidence intervals around the mean

When looking at search latency (Figure 5), we found a significant main effect of set size, with the critical object looked at earlier in set size 3 than 5 and 7. Moreover, there was a

significant main effect of target. The critical object was fixated earlier on target-present than target-absent trials. There was also a significant a main effect of semantic relatedness, whereby participants looked at the critical object earlier when it was semantically unrelated than related to the distractors. This was especially the case for set size 5 and 7 (for the two-way significant interaction of semantic relatedness and set size) and when the critical object was visually dissimilar to the distractors (for the two-way interaction of semantic relatedness and visual similarity). We also found significant two-way interactions between target and set

size, with the critical object fixated earlier on target-present than -absent trials, especially on set size 5 and 7; between visual similarity and target, whereby the critical was looked at earlier when it was visually dissimilar from the distractors, especially on target-present trials; and between visual similarity and set size, with shorter search latencies when the critical object and distractors were visually dissimilar, especially on set size 7. Finally, there was a

significant three-way interaction between semantic relatedness, target and set size. As set size increased from 3 to 7, the critical object was looked at earlier when it was semantically unrelated to the distractors, especially on target-absent trials (See Table 3 for the model output).

**Table 3:** Linear mixed-effects model output for search latency on the critical object

Dependent Variable	Predictor	β	SE	t-value	Pr ( > | t | )
Search Latency	Intercept	430.88	17.83	24.17	**< 0.001**
	Semantic Relatedness	138.78	31.01	4.48	**< 0.001**
	Set size (3 vs. 5)	138.80	25.24	5.50	**< 0.001**
	Set size (3 vs. 7)	281.90	25.29	11.15	**< 0.001**
	Target	- 73.68	25.11	- 2.93	**0.004**
	Visual Similarity	- 9.05	31.93	- 0.28	0.78
	Semantic Relatedness:Target	- 64.33	35.48	- 1.81	0.07
	Semantic Relatedness:Set size (3 vs. 5)	128.63	43.87	2.93	**0.004**
	Semantic Relatedness:Set size (3 vs. 7)	229.37	44.13	5.20	**< 0.001**
	Target:Set size (3 vs. 5)	- 85.19	35.78	- 2.38	**0.02**
	Target:Set size (3 vs. 7)	- 181.27	35.81	- 5.06	**< 0.001**
	Target:Visual Similarity	68.99	22.76	3.03	**0.003**
	Visual Similarity:Set size (3 vs. 5)	53.37	45.21	1.18	0.24
	Visual Similarity:Set size (3 vs. 7)	130.56	45.43	2.87	**0.004**
	Semantic Relatedness:Visual Similarity	83.23	36.43	2.29	**0.02**
	Semantic Relatedness:Target:Set size (3 vs. 5)	- 94.05	50.72	- 1.85	0.06
	Semantic Relatedness:Target:Set size (3 vs. 7)	- 123.91	51.03	- 2.43	**0.01**

*Note*. Predictors are listed in the table in the same order as they were entered in the model. The predictors were: target (absent = -.5, present = .5), semantic relatedness (unrelated = -.5, related = .5), visual similarity (dissimilar = -.5, similar = .5), and set size (3, 5, 7). Two planned comparisons were set for set size: 3 vs. 5 (3 = -.5, 5 = .5) and 3 vs. 7 (3 = -.5, 7 = .5).

**Fig. 6:**
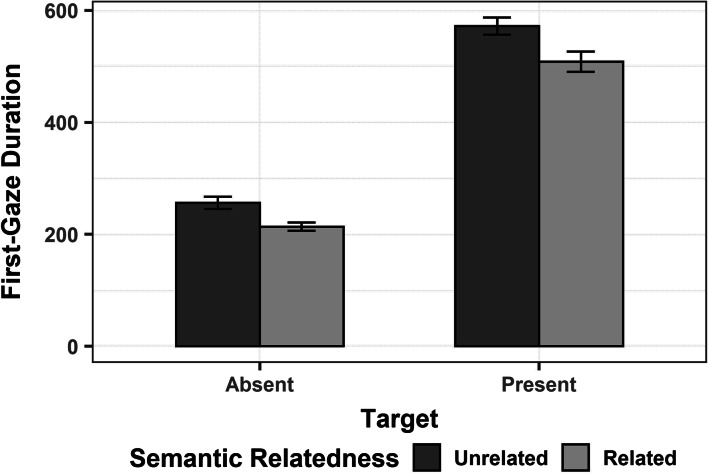
Mean first-gaze duration (ms) on the critical object on target-absent and -present trials (on the x-axis), in the semantically unrelated (dark grey) vs. related (light grey) condition. Error bars represent 95% confidence intervals around the mean

On first-gaze duration (Figure 6), there was a significant main effect of target, with the critical object fixated for longer on target-present than -absent trials. There was also a significant main effect of semantic relatedness: the critical object was fixated less when semantically related than unrelated to the distractors (See Table 4 for the model output).

**Table 4:** Linear mixed-effects model output for first-gaze duration on the critical object

Dependent Variable	Predictor	β	SE	t-value	Pr ( > | t | )
First-gaze Duration	Intercept	387.67	7.12	54.44	**< 0.001**
	Target	306.91	13.27	23.13	**< 0.001**
	Semantic Relatedness	- 51.70	8.45	- 6.12	**< 0.001**
	Visual Similarity	- 18.77	9.53	- 1.97	0.05
	Semantic Relatedness:Target	- 25.16	13.29	- 1.89	0.06
	Target:Visual Similarity	- 29.08	15.94	- 1.82	0.07

*Note*. Predictors are listed in the table in the same order as they were entered in the model. The predictors were: target (absent = -.5, present = .5), semantic relatedness (unrelated = -.5, related = .5), and visual similarity (dissimilar = -.5, similar = .5).

Interestingly, the effect of semantic relatedness on first-gaze duration was also found when looking only at the trials in which the critical object was also the first fixated object after array onset (i.e., 985 trials)^8^. This finding contrasts with Henderson et al. (1987) who showed that a critical object receives shorter fixations only when a semantically related object is looked at before it.

## Discussion

The present visual search study provides fresh evidence that object semantics are processed in extra-foveal vision and used to guide early overt attention. In our task, participants were cued with a target to search in object arrays of different sizes (3, 5, 7), which displayed a critical object and other distractors. The critical object was either the search target (present trials), or a target’s semantically related competitor (absent trials), either salient or non-salient, and it was either semantically related or unrelated to the distractors, which were always semantically related to each other. We found that the critical object was more likely to attract the very first fixation after the onset of the object array, and overall be inspected for the first time earlier, when semantically unrelated than related to the distractors, especially on target-absent trials.

Our findings clearly indicate that object semantic information can be extracted in extra-foveal vision, as early as the onset of the visual context, to guide early overt attention.

Moores et al. (2003) and Belke et al. (2008) had also reported semantic relatedness effects on initial eye movements during search. However, these earlier studies have been criticised by Daffron and Davis (2016) who suggested that effects of semantic relatedness might have been confounded by the repeated presentation of the visual stimuli to the participants. Thus, overt attention might have been biased by remembered visual features of the objects, rather than by their semantic features. The same criticism cannot be raised against our study, where each object was never presented more than once to each participant. In contrast with de Groot et al. (2016) who found a stronger effect of visual similarity on early overt attention compared to semantic relatedness (but see Nuthmann et al., 2019 for a re-analysis showing a reduced primacy), our study did not find any significant main effect of visual similarity on eye-movement behaviour. Nevertheless, we observed significant interactions between visual similarity and semantic relatedness on response times and search latencies. These results seem to suggest that, as visual search unfolds, the visual similarity between objects is accessed to refine the ongoing semantic guidance and optimises visual search. We believe that this suggestion is strengthened by the evidence that the effect of semantic relatedness on search guidance was weaker on target-present than -absent trials. When the target is present, participants might rely more on visual information to facilitate search, thus reducing the effect of semantic relatedness on eye movements (Huettig & Altmann, 2005; Huettig & Mcqueen, 2007).

The evidence of semantic relatedness effects on the very first fixations may suggest that extra-foveal semantic processing relies on a global deployment of covert attention, i.e., distributed attention, occurring across the visual field (e.g., Treisman, 2006). Such processing eases a rapid extraction of the general layout of the information within the context, i.e., its gist, as well as the objects therein, including some summary statistics, i.e., ensemble perception (see Whitney & Leib, 2018 for a recent review); both in naturalistic scenes (Davenport, 2007; Gordon, 2004) or object arrays (Auckland et al., 2007; Starreveld, Theeuwes, & Mortier, 2004). Alternatively, observers might be able to access partial semantic information of an object, such as its category membership, through a rapid and parallel processing of disjunctive sets of visual features characterizing that category (Evans & Treisman, 2005), which would occur pre-attentively across the visual field (Treisman, 2006; see also J. M. Wolfe & Utochkin, 2019, for a very recent discussion about pre-attentive features). For example, observers might detect the presence of a four-footed animal by using specific feature detectors: eyes, a set of legs, head, fur. These feature detectors would mediate the classification/categorization of both natural (e.g., animal) and non-natural (e.g., vehicle) objects but not their full identification, which would still require the serial deployment of overt attention to bind their features together.

Other strands of research in vision science also converge on the evidence that a great deal of information, including high-level conceptual information, is available in extra-foveal vision, either covertly or pre-attentively, prior to the first deployment of overt attention. Saccadic programming is, for example, facilitated when attention is covertly deployed to the target object (e.g., Kowler, Anderson, Dosher, & Blaser, 1995), and its recognition enhanced when the target is crowded by other objects (e.g., Harrison, Mattingley, & Remington, 2013; B. A. Wolfe & Whitney, 2014). Such facilitation may also depend on predictive remapping (e.g., Hall & Colby, 2011; Higgins & Rayner, 2014), which makes available a rich set of visual features, including object-selective information (e.g., for an example on faces, see B. A. Wolfe & Whitney, 2015), from peripheral vision prior to saccadic eye-movements (e.g., Melcher, 2007). If object-selective information can be accessed in extra-foveal vision, it can be then used to guide saccadic programming in a top-down manner (Moores et al., 2003). That is, overt attention would be allocated onto regions of the visual context sharing semantic features with the search target, and hence more likely to contain it. As our study also shows, when the critical object is semantically related to a visual context of semantically homogeneous distractors, its recognition is delayed.

In fact, a methodological novelty of this study is precisely that all distractor objects were always semantically related (hence homogenous, e.g., a set of vehicles) in the visual context. We assumed that search efficiency was not only influenced by the semantic relatedness between the target and the distractors (as in previous studies, Belke et al., 2008; de Groot et al., 2016; Moores et al., 2003; Nuthmann et al., 2019), but also by the semantic relatedness of the distractors themselves. This manipulation of the visual context, which substantially departs from previous research (e.g., de Groot et al., 2016; Nuthmann et al., 2019), increased the guidance of early overt attention exerted by the extra-foveal processing of object semantics. In fact, when the critical object was semantically related to the distractors (e.g., a car), all objects equally competed for visual attention. When the critical object was instead semantically unrelated (e.g., a fork) to the semantically homogeneous distractors, there was no competition thus boosting its identification in extra-foveal vision. Future research is needed to better describe how the semantic relatedness between distractors impact on visual search, for example, by systematically varying their semantic relatedness on a continuum, i.e., from distractors all semantically homogeneous (i.e., the current study) to distractors that are all heterogeneous.

Our results also seem to suggest that some parallel processing may occur (Buetti, Cronin, Madison, Wang, & Lleras, 2016), but as semantic information may be only partially acquired in extra-foveal vision (Gordon, 2004), such processing may be limited in nature. In fact, if the semantics of all objects are processed in parallel across the visual field, i.e., regardless of the number of distractor objects, then we should observe an identical processing advantage of the critical object when semantically unrelated than related to the semantically homogenous distractors. That is, our data should have shown, for example, exactly the same immediate probability that the critical object is looked at first across arrays of increasing size when unrelated to the distractor objects, i.e., a canonical “pop-out” effect. We observed instead that the probability of immediate fixation to the critical object decreases with the increasing of the set size for both semantically unrelated and related critical objects. This finding would be difficult to account for in terms of full parallel processing, as we would have to assume that the processing of high-level semantic information of all objects should be completed immediately after the onset of the array. Nevertheless, if observers only processed in extra-foveal vision the semantics of just one object, i.e., serial processing, then the prioritization of a semantically unrelated object should be substantially reduced (and perhaps become indistinguishable) compared to a semantically related object as the number of semantically homogenous distractors increases. Instead, even as the identification of the critical object became harder as the number of distractors increased, a semantically unrelated object maintained an advantage to be prioritized over a semantically related object. Moreover, a further analysis of the latency to generate the very first saccade after the onset of the object array, which reflects the time to select the first target candidate (Malcolm & Henderson, 2009, 2010), showed no effect of set size^9^; which also counters a serial processing account of object semantics. In fact, if participants had covertly and serially attended to all the objects in the array prior to making the first fixation, then the first saccade should have occurred later for arrays of increasing size.

Semantic processing also mediated the time spent looking at the critical object when it was foveated for the first time. In particular, we found that the critical object was fixated for the first time less when it was semantically related than unrelated to the distractors, also when restricting the analysis to the trials where the critical object was the first object fixated. This finding extends the observation of Henderson et al. (1987) on a memory recognition task to a visual search task. According to these authors, the facilitated processing for a semantically related object arises from object-to-object priming, whereby a critical object is more readily identified when a semantically related object is fixated immediately before it. The current study adds to this finding that the semantic information of more than a single object was accessed, possibly covertly, in extra-foveal vision during the very first fixation, in line with previous research on object arrays (Auckland et al., 2007) or naturalistic scenes (Davenport, 2007) and affected early overt attention. In fact, only if the semantics of the objects are immediately available at the onset of the array, we can observe effects of semantic relatedness on the duration of the very first fixation.

It is important to note that we did not find effects of visual saliency on any of the measures examined. This result is consistent with previous research showing that low-level visual saliency is not an influential factor in the guidance of overt attention during visual search on object arrays (Chen & Zelinsky, 2006).

The current study demonstrates an extra-foveal processing of object semantics using object arrays, as early as at the onset of the visual context, but such a finding is much more controversial when naturalistic scenes are used. On one hand, some studies have shown that there is no difference in the speed of target identification due to object semantics, and only fixation measures of object inspection are modulated by it (De Graef, Christiaens, & D’Ydewalle, 1990; Henderson, Weeks, & Hollingworth, 1999; Spotorno & Tatler, 2017; Võ & Henderson, 2011), which suggests that semantic information cannot be processed in extra-foveal vision to a degree that can influence the allocation of early overt attention. On the other hand, some studies have reported that inconsistent objects are fixated earlier and for longer than consistent objects (LaPointe & Milliken, 2016; Loftus & Mackworth, 1978) both by younger and older adults (e.g., Borges, Fernandes, & Coco, 2019), and elicit a larger negative shift of fixation-related potential activity in the fixation preceding the target fixation (Coco, Nuthmann, & Dimigen, 2019); altogether, these findings support an extra-foveal processing of object semantics, and consequently the early capture of overt attention. Possibly, the inconsistencies across studies are due to the fact that naturalistic scenes vary along a wider range of low- and high-level features than object arrays. For example, objects in a naturalistic scene (e.g., a restaurant) are usually arranged according to semantic (e.g., a chair is a common object in a restaurant scene, whereas a bed would be inconsistent with it) and syntactic (e.g., a chair does not fly) information (Biederman, 1976; Draschkow & Võ, 2017; J. M. Wolfe, Võ, Evans, & Greene, 2011). Moreover, in a single glance, observers can pre-attentively accrue a considerable amount of global information about a scene (e.g., its semantic category, Greene & Oliva, 2009; Oliva & Torralba, 2006). Observers may therefore integrate object-specific information with global scene information to optimise search (Castelhano & Heaven, 2011; Castelhano & Henderson, 2007; Malcolm & Henderson, 2010; Neider & Zelinsky, 2006); but see Greene and J. M. Wolfe (2011) showing that global scene information does not seem to improve visual search. Moreover, the position of an object relative to the centre of the screen, as well as the global and local crowding surrounding it, may reduce its extra-foveal processing (Pelli, 2008; Pelli, Palomares, & Majaj, 2004; Rosenholtz, 2016). So, future research is needed to investigate, more systematically, the low- and high-level features that are truly processed in extra-foveal vision regardless of whether the visual context is an object array or a naturalistic scene.

In sum, our findings suggest that object semantics can be processed in extra-foveal vision as early as at the onset of the visual context, and play a primary and predominant role on guiding early overt attention, above and beyond other factors such as low-level visual saliency and visual similarity, at least in the context of the current study. Our study, thus, critically contributes to the debate around the influence of semantic information on eye movement guidance, and on its temporal dynamics.

### Electronic supplementary material

ESM 1 (DOCX 28.9 kb)

ESM 2 (DOCX 5.80 mb)

ESM 3 (DOCX 33.6 kb)

## Appendix A: Model selection procedure

We used a forward best-path model selection technique to define the fixed and the random effect structures of our models (see Barr, Levy, Scheepers, & Tily, 2013; Coco, Malcolm, & Keller, 2014, for examples of this approach). We started with a basic model including participant and item as random intercepts (i.e., DV ~ (1 | participant) + (1 | item) in Wilkinson notation). Then, we added each fixed effect to this basic model, individually (e.g., DV ~ semantic relatedness + (1 | participant) + (1 | item)). Such model was then compared with the same model but now including either correlated (e.g., DV ~ semantic relatedness + (1 + semantic relatedness | participant) + (1 | item)) or uncorrelated random slopes (e.g., DV ~ semantic relatedness + (1 | participant) + (1 | item) + (0 + semantic relatedness | participant)). We then compared the three models on their log-likelihood using chi-square tests. We retained the model with the strongest fit (lowest p-value with a threshold of p < 0.09 to include marginally significant results). We repeated the same procedure independently for each fixed effect, and ordered their inclusion based on their log-likelihood significant fit (e.g., if semantic relatedness gave us better fit than visual similarity, we included semantic relatedness before visual similarity), whereas non-significant fixed effects were dropped. Finally, we added interactions, but only for those fixed effects that were retained during model selection.

## Footnotes

1 We conceived our initial design to replicate the results by de Groot et al. (2016), who used target-absent experimental trials and target-present filler trials. We run this experimental design on the first 72 participants (24 for each set size, i.e., 3, 5, 7). Then, we decided to test 72 further participants on the opposite scenario (i.e., target- present experimental trials and target- absent filler trials) to investigate whether the results would corroborate. In the analysis, we compare the data of target-absent vs. target-present within the same model as a between-participant variable to increase our statistical sample and examine whether effects of semantic relatedness vary according to the presence of the target.

2 We created the experimental arrays for the three different set size conditions starting with the 3-object arrays (e.g., car - truck - motorcycle) and then adding two more objects (e.g., helicopter - bus) to obtain the 5-object arrays, and two more (e.g., boat - train) for the 7-object arrays. Then, we manipulated set size between-participant, to avoid repeating the same visual objects across trials and increase the number of observations for each set size.

3 The critical object in the array is the one for which we modified the visual saliency and its semantic relatedness with respect to the other distractor objects. The critical objects were selected prior to data collection and data analyses, especially taking into account the effectiveness and easiness of the visual saliency manipulation. We realise that in the target-absent trials for the semantic-related condition, all objects in the display could be critical objects, but effectively, there is always only one object in the array on which the visual saliency manipulation was carried out. Moreover, the same critical objects have to be chosen between target-present and target-absent trials to compare eye-movement responses on an equal ground.

4 We preferred to use words rather than pictures as cues to avoid participants using high-level visual information to search for the target within the object array.

5 The size of the fixation cross was 42 x 42 pixels (1.20° x 1.21° of visual angle) and it was surrounded by an invisible bounding box of 70 x 70 pixels (1.96° x 1.96° of visual angle) that had to be looked at for 100 ms to trigger the presentation of the object array, i.e., the beginning of the trial.

6 We regarded as accurately responding to a target-present trial when both the search response and the number response were accurate.

7 Semantic relatedness (unrelated: *M* = .41, *SD* = .49; related: *M* = .27, *SD* = .45; *β* = - .76, *SE* = .13, *z* = - 5.93, *p*< .001); Set Size 3 vs. 5 (3: *M* = .43, *SD* = .50 ; 5: *M* = .32, *SD* = .47; *β* = - .56, *SE* = .16, *z* = - 3.49, *p*< .001); Set Size 3 vs.7 (7: *M* = .25, *SD* = .43; *β* = - .99, *SE* = .17, *z* = - 6.01, *p*< .001); Target (absent: *M* = .31, *SD* = .46; present: *M* = .38, *SD* = .49; *β* = .43, *SE* = .10, *z* = 4.33, *p*< .001). OP*u* for each set size (3: *M* = .50, *SD* = .50; 5: *M* = .41, *SD* = .49; 7: *M* = .30, *SD* = .46) was significantly higher than CP (all *ps*<.001), whereas OP*r* did not differ significantly from CP, across all set sizes (3: *M* = .36, *SD* = .48; 5: *M* = .24, *SD* = .42; 7: *M* = .19, *SD* = .40)(all *ps*>.05).

8 Target (absent: *M* = 238.93, *SD* = 150.04; present: *M* = 558.29, *SD* = 282.87; *β* = 322.29, *SE* = 17.75, *t* = 18.16, *p*< .001); Semantic relatedness (unrelated: *M* = 445.01, *SD* = 286.29; related: *M* = 374.09, *SD* = 271.05; *β* = - 86.44, *SE* = 15.48, *t* = - 5.58, *p*< .001).

9 The time needed to make the first saccade did not increase as the number of the objects in the array increased from 3 (*M* = 156.28, *SD* = 56.37) to 5 (*M* = 147.01, *SD* = 47.33)(*β* = - 8.80, *SE* = 5.86, *t* = - 1.50, *p* = .14), and to 7 (*M* = 154.27, *SD* = 55.51)(*β* = - 2.41, *SE* = 5.86, *t* = - .41, *p* = .68).
