# Supplementary material for: Extra-foveal Processing of Object Semantics Guides Early Overt Attention During Visual Search
Source: Atten Percept Psychophys. 2019 Dec 2;82(2):655–70. doi: 10.3758/s13414-019-01906-1 (PMC7246246; doi:10.3758/s13414-019-01906-1)
Supplement: Supplementary file 1 — (DOCX 28.9 kb) [file 13414_2019_1906_MOESM1_ESM.docx]

**Supplemental Material A**

**Online Survey and Stimuli Norms**

A total of 387 object pictures were selected from the BOSS database^[[1]](#footnote-1)^ (Brodeur, Dionne-Dostie, Montreuil, & Lepage, 2010; Brodeur, Guérard, & Bouras, 2014), scaled from their original size (2000 x 2000 pixels) to 150 x 150 pixels, and pre-tested through an online survey administered by using the Online Surveys tool. Ten participants (3 female), ranging between 23 and 63 years of age (*M* = 31.40, *SD* = 12.85) and 11 and 18 years of schooling (*M* = 16.6, *SD* = 2.72), took part in the survey. All reported being native English speakers, with English (either British or American) as first language.

The survey was divided in three parts. For each part of the survey, a random sequence of 129 object pictures was generated. The order of presentation did not differ across participants. In the survey, participants were presented with one object at the time and asked to name them and to determine the semantic category they belonged to by making a selection across 20 given semantic category labels. We took six category labels from the Battig and Montague’s (1969) category norms, and two from the Brodeur et al.'s (2014) normative study. Additionally, 12 new category labels were originated here (Table S1 shows the 20 semantic category labels and their source). For both the naming and the semantic categorization task, instructions were almost identical to those used in the BOSS normative studies (Brodeur et al., 2010, 2014) and were given both prior to the beginning of the survey and upon presentation of each object. In the naming task, participants were asked to identify each object by typing the first name that came to mind. In case they could not recognize the object, they were instructed to type “DKO” (Do not Know Object). If they could recognize the object but they did not know its name, they typed “DKN” (Do not Know Name), whereas they typed “TOT” (Tip of the Tongue) in case they could not retrieve the name of the object in that moment. In the semantic categorization task, they were instructed to determine the semantic category the object belonged to, by making a selection across 20 given categories and an “Others” choice. The “Others” choice had to be picked either if no category seemed to be appropriate or in case a DKO response had been previously provided in the naming task.

For each object, the name and the category provided by participants were computed in order to extract modal name, modal category and the corresponding percentages of agreement. The modal name consisted in the name given by the highest percentage of participants for a given object after the exclusion of the DKO, DKN and TOT responses. This percentage corresponded to the modal name agreement which indicated to what extent participants agreed to use the modal name to identify the object. The same procedure was used to obtain the modal category and the modal category agreement. Of the 387 objects initially submitted to the online survey, 47 were excluded either because they had a DKO, DKN, or TOT score over 20%; their model name was incorrect or, when correct, had a percentage of agreement of less than 20%; they were not assigned to any specific category (“Others” as modal category); or because their modal category agreement was below 50%. Interestingly, the 90.88% of the remaining 340 objects had the same modal name in both the online survey and the BOSS normative study (Brodeur et al., 2010, 2014). Hence, when the modal name differed (e.g., “phone” and “motorbike” in the online survey vs. “telephone” and “motorcycle” in the BOSS norms), we decided to label the objects by using the BOSS modal names. This allowed us to apply two additional selection criteria to our stimulus set: (1) We excluded objects with either a BOSS modal name agreement below 50%, or a DKO, DKN or TOT score over 20% (2) in case two or more objects were given the same modal name and modal category, we included in the stimulus set only the object with the highest BOSS modal name agreement. Eventually, the stimulus set resulted in a total of 289 object pictures, of which only 224 were used as experimental stimuli. Table S2 presents the descriptive statistics for the variables examined on the stimulus set.

**References**

Battig, W. F., & Montague, W. E. (1969). Category norms of verbal items in 56 categories A replication and extension of the Connecticut category norms. *Journal of Experimental Psychology*, *80*(3), 1–46. https://doi.org/10.1037/h0027577

Brodeur, M. B., Dionne-Dostie, E., Montreuil, T., & Lepage, M. (2010). The Bank of Standardized Stimuli (BOSS), a New Set of 480 Normative Photos of Objects to Be Used as Visual Stimuli in Cognitive Research. *PLoS ONE*, *5*(5), e10773. https://doi.org/10.1371/journal.pone.0010773

Brodeur, M. B., Guérard, K., & Bouras, M. (2014). Bank of Standardized Stimuli (BOSS) Phase II: 930 New Normative Photos. *PLoS ONE*, *9*(9), e106953. https://doi.org/10.1371/journal.pone.0106953

Table S1

*The 20 Semantic Category Labels Grouped by Source*

| No. | Semantic category label | Source |
| --- | --- | --- |
| 1 | Four-footed animal |  |
| 2 | Fruit |  |
| 3 | Musical instrument | Battig and Montague (1969) |
| 4 | Vegetable |  |
| 5 | Insect |  |
| 6 | Bird |  |
| 7 | Weapon and war related item | Brodeur et al. (2014) |
| 8 | Body part |  |
| 9 | Aquatic animal |  |
| 10 | Electrical appliances |  |
| 11 | Games and toys |  |
| 12 | Home furnishings |  |
| 13 | Personal accessories | Current study |
| 14 | Personal care product |  |
| 15 | Hand labour tool |  |
| 16 | Item of clothing |  |
| 17 | Kitchen item |  |
| 18 | Means of transportation/Vehicle |  |
| 19 | Sport equipment |  |
| 20 | Stationery supplies |  |

Table S2

*Minimum, Maximum, Mean, and Standard Deviation (%) for the Stimulus Set*

| Variables | Minimum | Maximum | Mean | SD |
| --- | --- | --- | --- | --- |
| Modal name agreement | 30 | 100 | 86.97 | 17.12 |
| DKO | 0 | 20 | 1.45 | 4.16 |
| DKN | 0 | 20 | 0.66 | 2.87 |
| TOT | 0 | 20 | 0.59 | 2.76 |
| Modal category agreement | 50 | 100 | 87.92 | 15.72 |

*Note.* DKO = Do not know object; DKN = Do not know name; TOT = Tip of the tongue.

1. In selecting the object pictures from the BOSS database, we excluded those with either a DKO, DKN, or TOT score over 25%; those with a modal name agreement below 45%; and/or those which could not be intuitively classified within the semantic category labels used in the current study. [↑](#footnote-ref-1)
