# Supplementary material for: Extra-foveal Processing of Object Semantics Guides Early Overt Attention During Visual Search
Source: Atten Percept Psychophys. 2019 Dec 2;82(2):655–70. doi: 10.3758/s13414-019-01906-1 (PMC7246246; doi:10.3758/s13414-019-01906-1)
Supplement: Supplementary file 2 — (DOCX 5.80 mb) [file 13414_2019_1906_MOESM2_ESM.docx]

**Supplemental Material B**

**Miniatures of the Experimental Arrays for Each Set Size by Semantic Relatedness and Visual Saliency Condition**


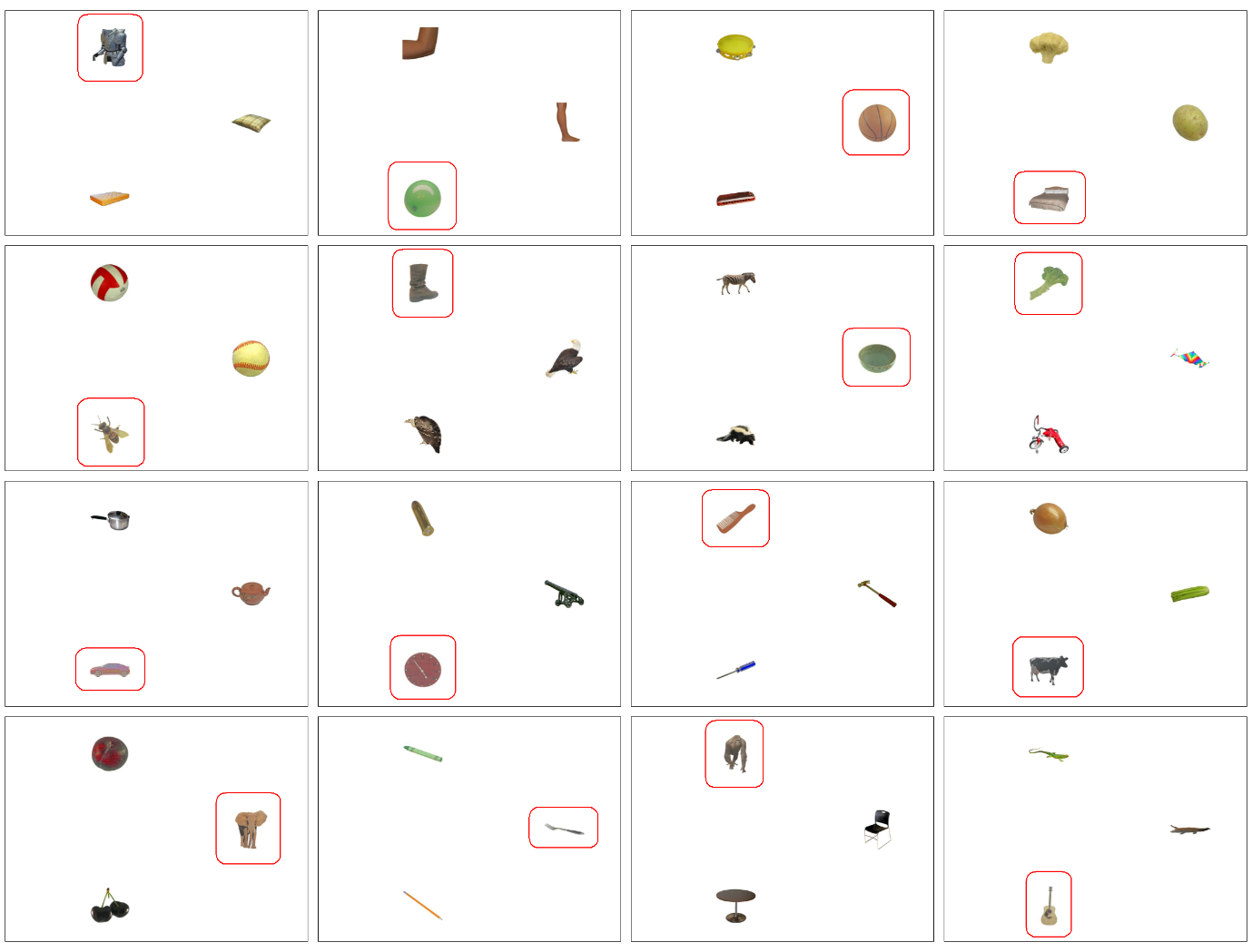


*Figure S1.* Experimental arrays with the critical objects (surrounded by their bounding boxes, in red) and distractors in the unrelated and non-salient conditions, for set size 3 (16 arrays out of 32).

*Supplemental material continues*


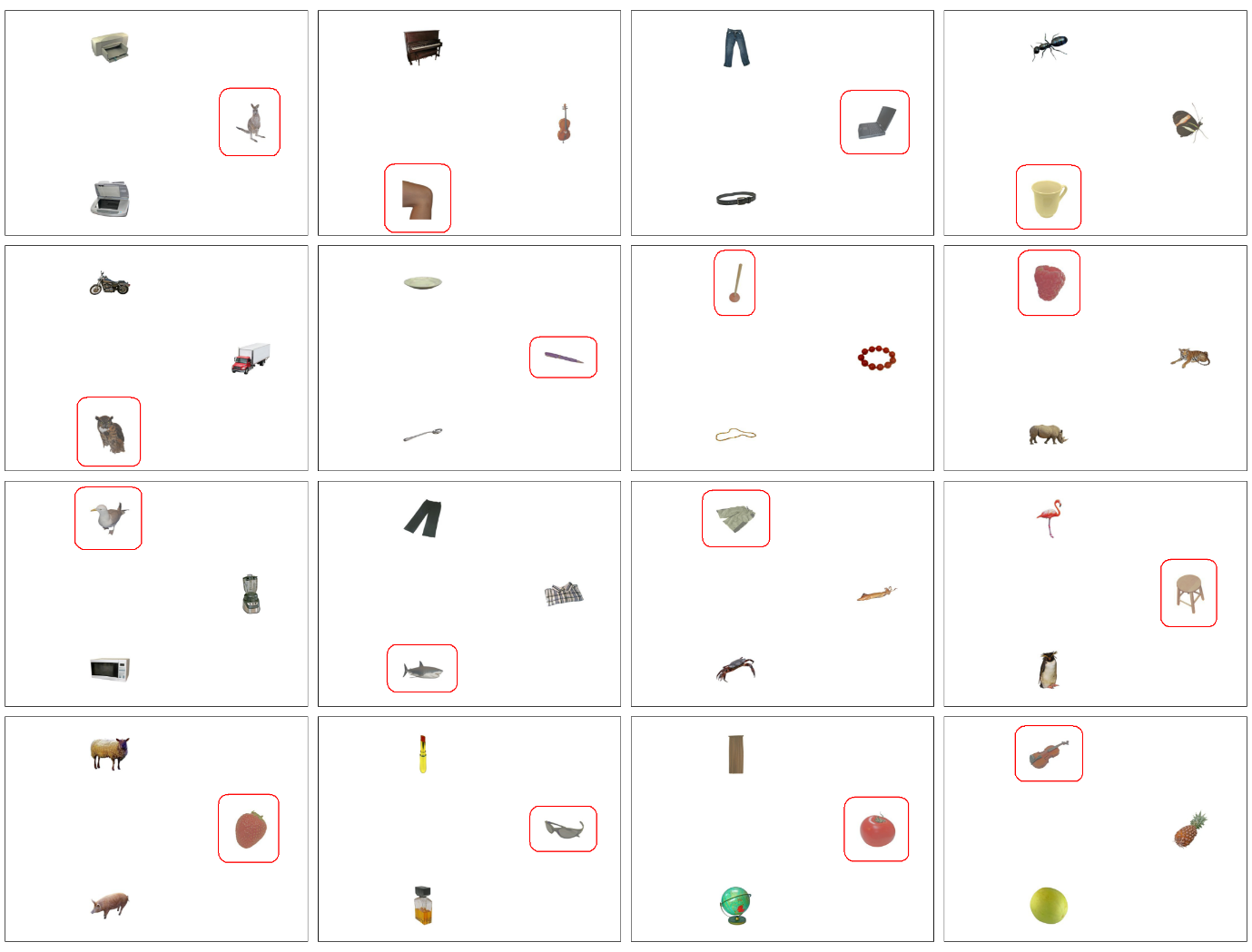


*Figure S2.* Experimental arrays with the critical objects (surrounded by their bounding boxes, in red) and distractors in the unrelated and non-salient conditions, for set size 3 (32 arrays out of 32).

*Supplemental material continues*


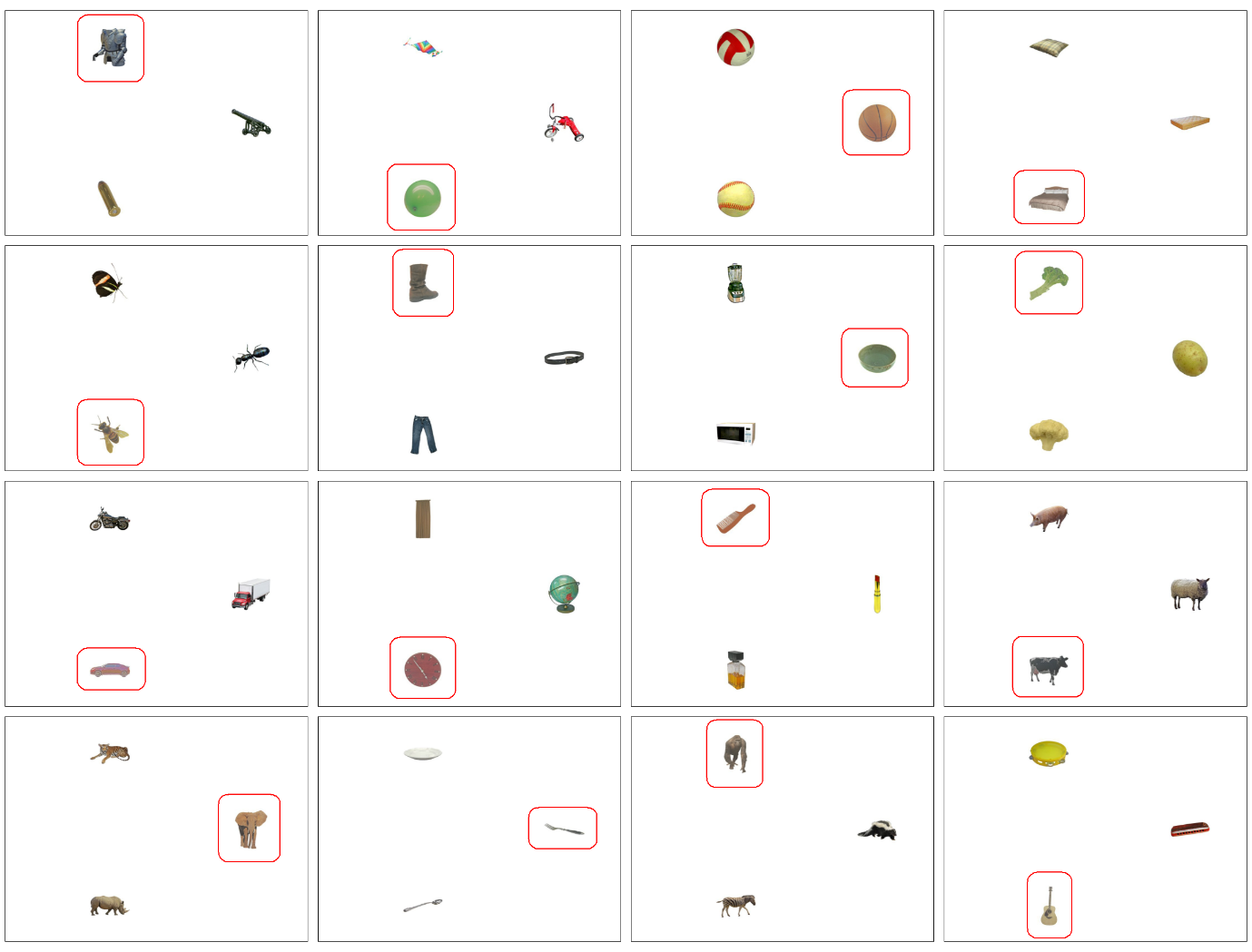


*Figure S3.* Experimental arrays with the critical objects (surrounded by their bounding boxes, in red) and distractors in the related and non-salient conditions, for set size 3 (16 arrays out of 32).

*Supplemental material continues*


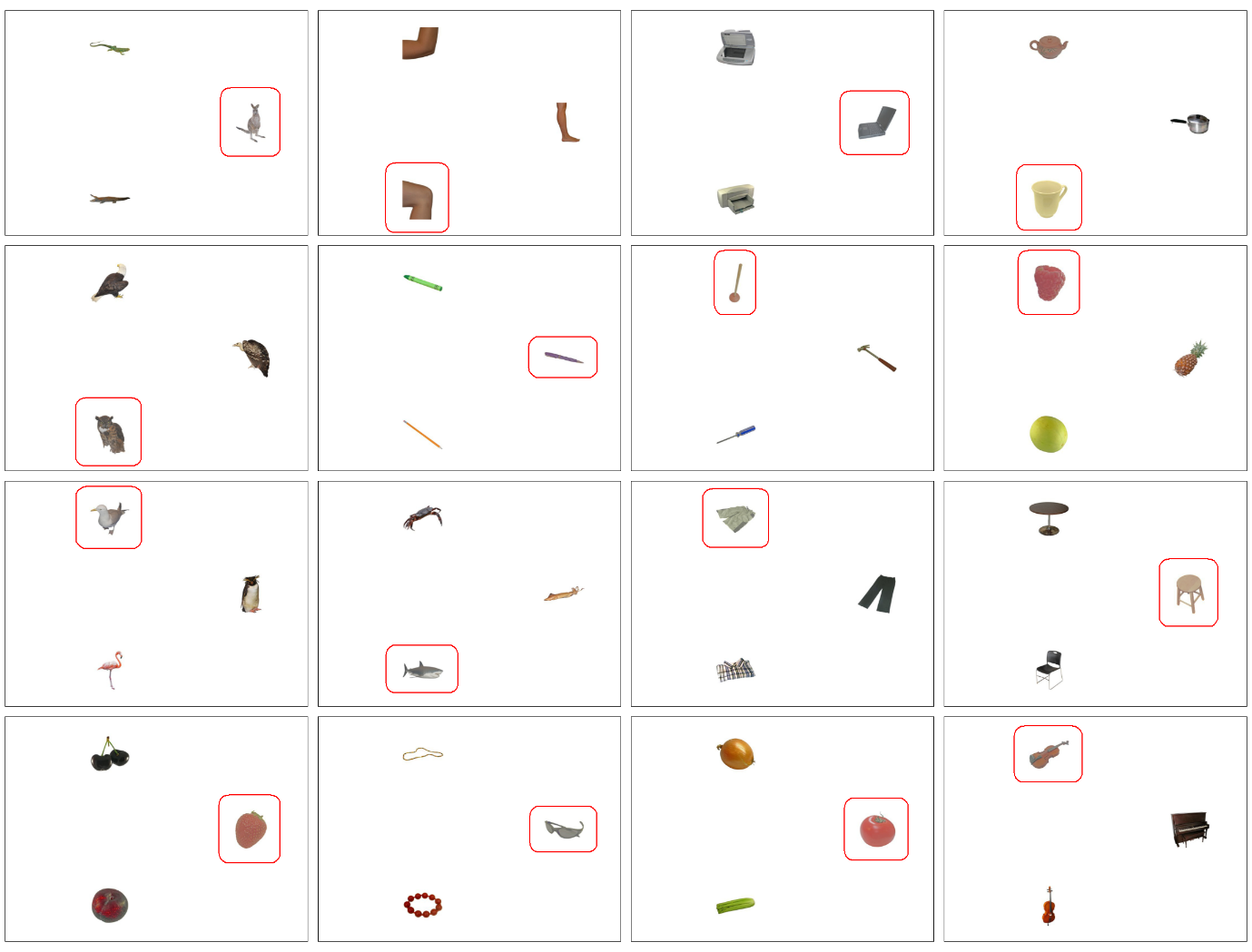


*Figure S4.* Experimental arrays with the critical objects (surrounded by their bounding boxes, in red) and distractors in the related and non-salient conditions, for set size 3 (32 arrays out of 32).

*Supplemental material continues*


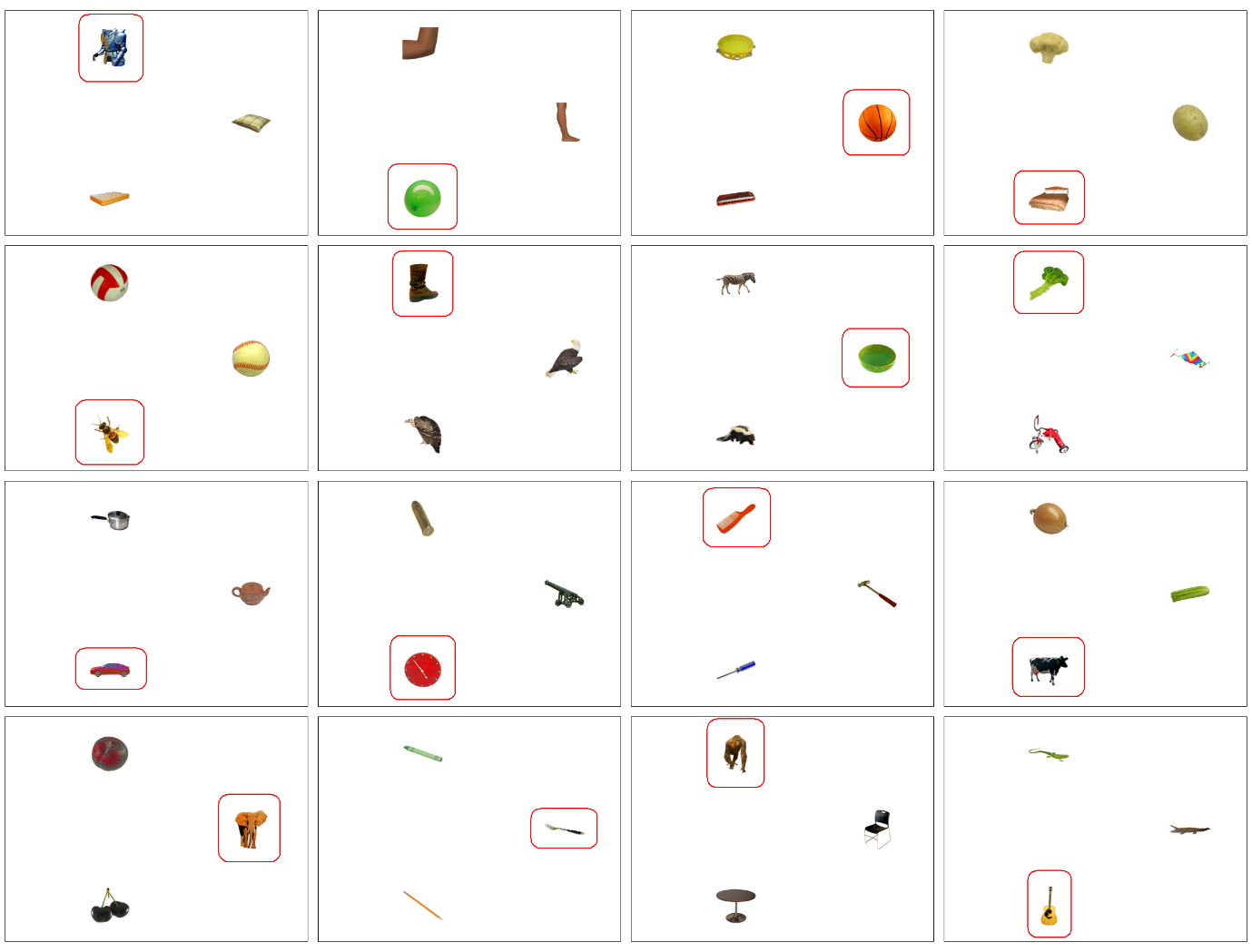


*Figure S5.* Experimental arrays with the critical objects (surrounded by their bounding boxes, in red) and distractors in the unrelated and salient conditions, for set size 3 (16 arrays out of 32).

*Supplemental material continues*


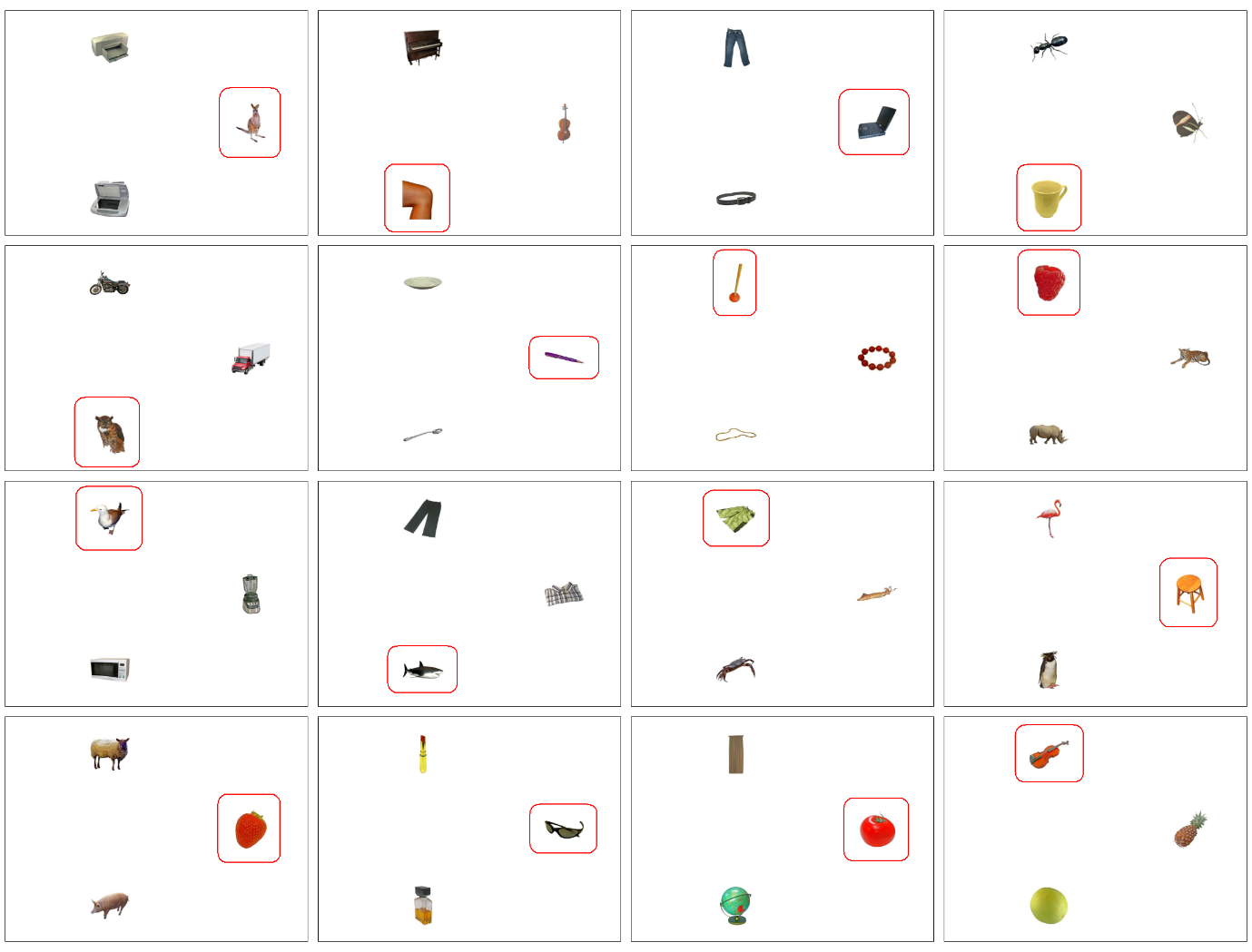


*Figure S6.* Experimental arrays with the critical objects (surrounded by their bounding boxes, in red) and distractors in the unrelated and salient conditions, for set size 3 (32 arrays out of 32).

*Supplemental material continues*


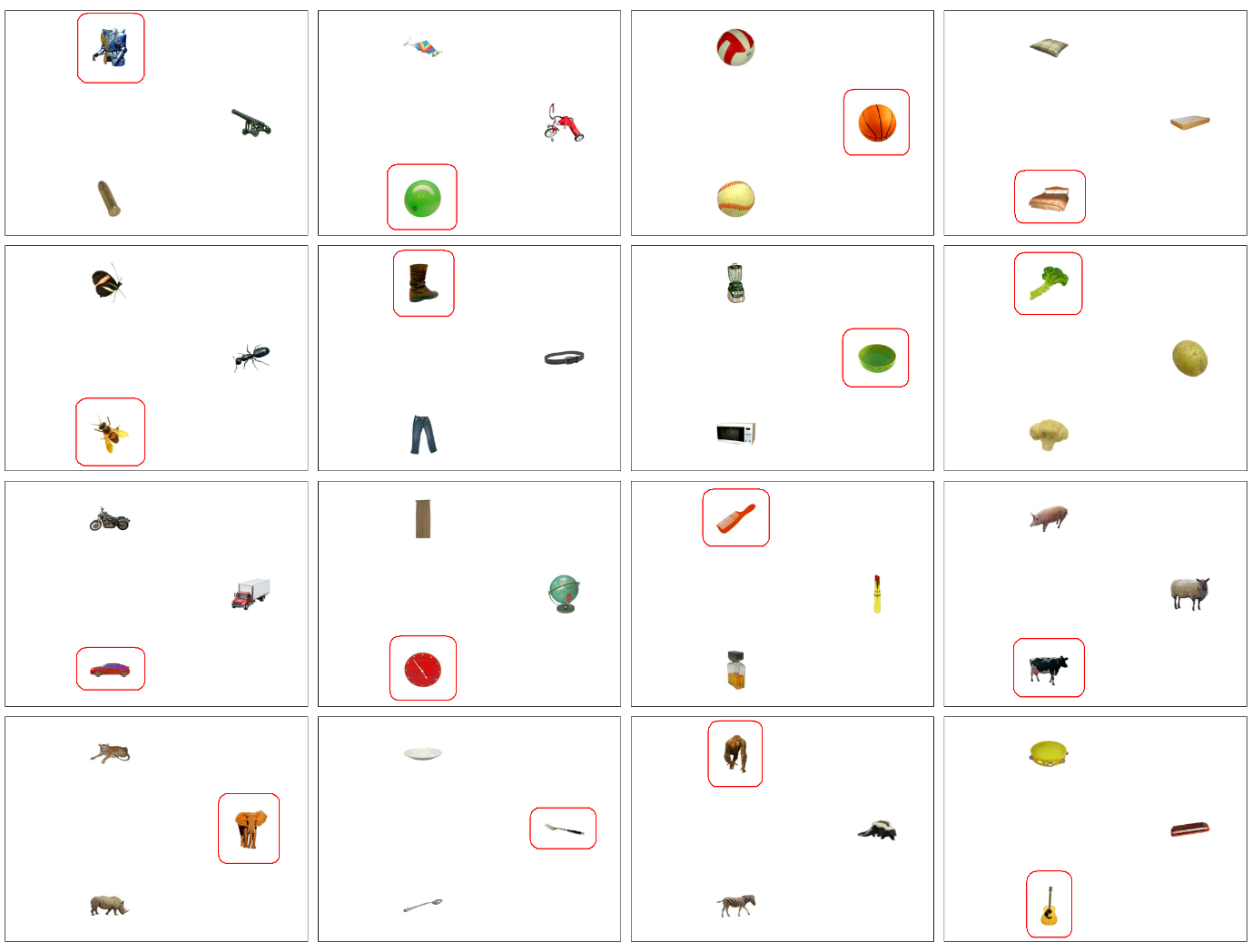


*Figure S7.* Experimental arrays with the critical objects (surrounded by their bounding boxes, in red) and distractors in the related and salient conditions, for set size 3 (16 arrays out of 32).

*Supplemental material continues*


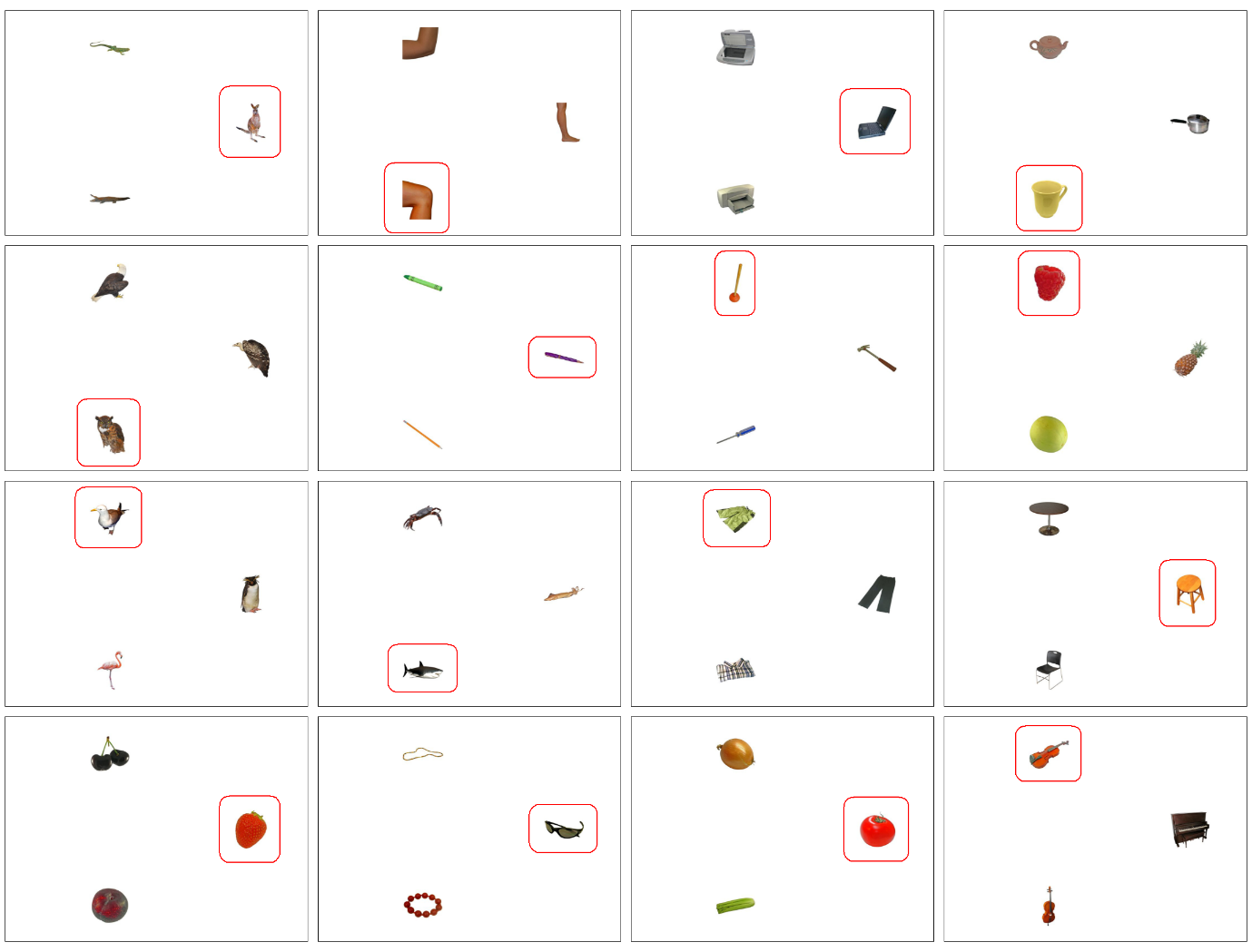


*Figure S8.* Experimental arrays with the critical objects (surrounded by their bounding boxes, in red) and distractors in the related and salient conditions, for set size 3 (32 arrays out of 32).

*Supplemental material continues*


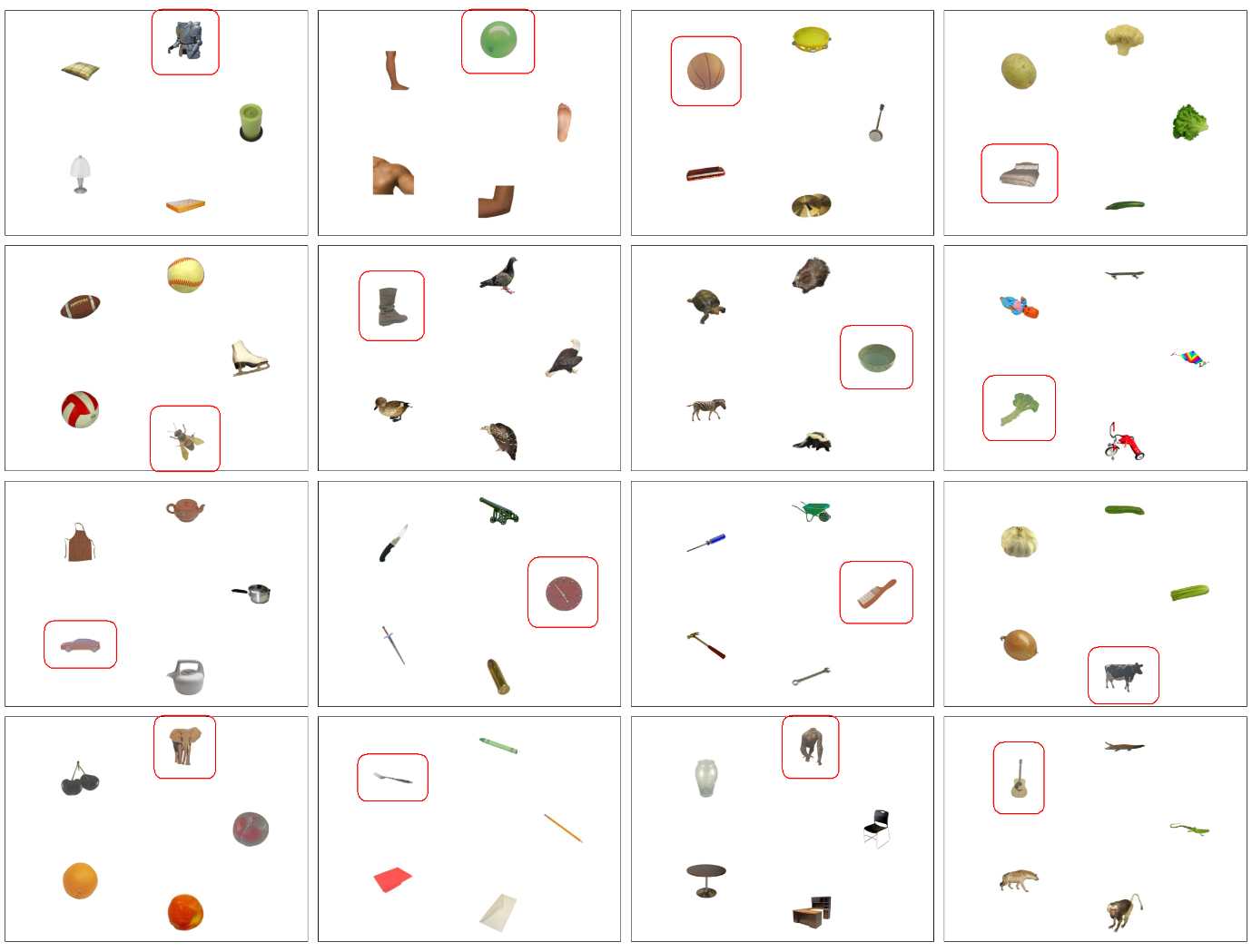


*Figure S9.* Experimental arrays with the critical objects (surrounded by their bounding boxes, in red) and distractors in the unrelated and non-salient conditions, for set size 5 (16 arrays out of 32).

*Supplemental material continues*


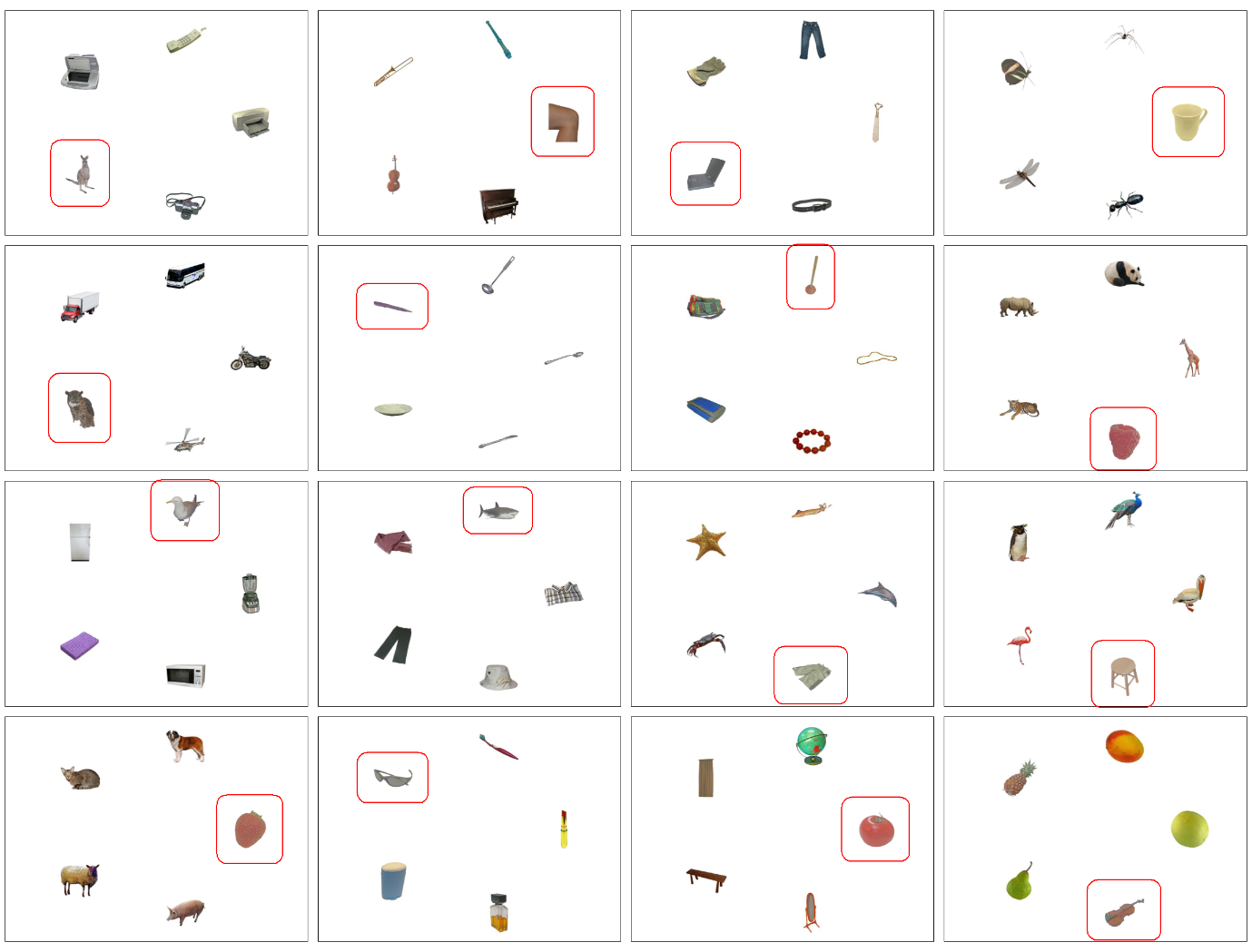


*Figure S10.* Experimental arrays with the critical objects (surrounded by their bounding boxes, in red) and distractors in the unrelated and non-salient conditions, for set size 5 (32 arrays out of 32).

*Supplemental material continues*


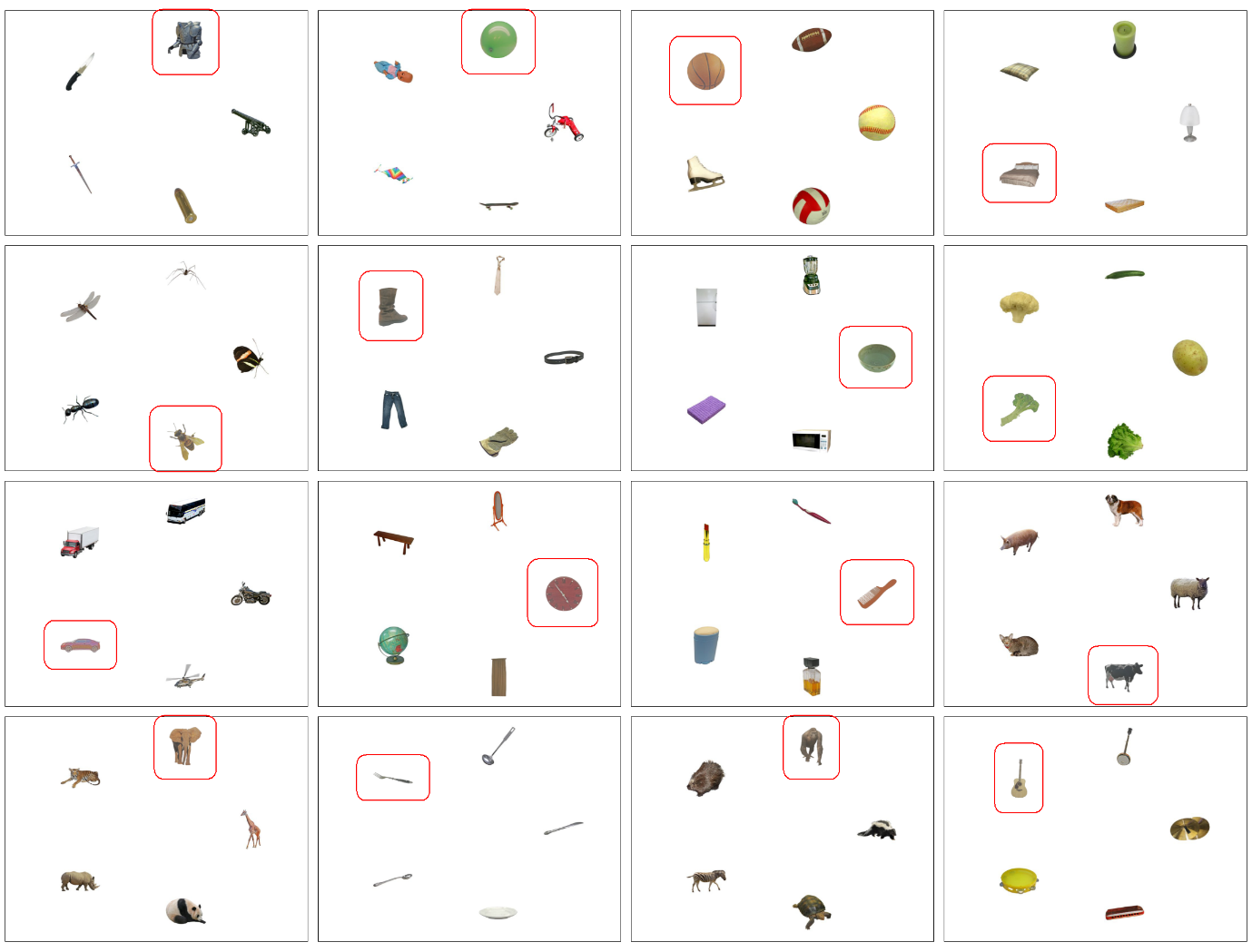


*Figure S11.* Experimental arrays with the critical objects (surrounded by their bounding boxes, in red) and distractors in the related and non-salient conditions, for set size 5 (16 arrays out of 32).

*Supplemental material continues*


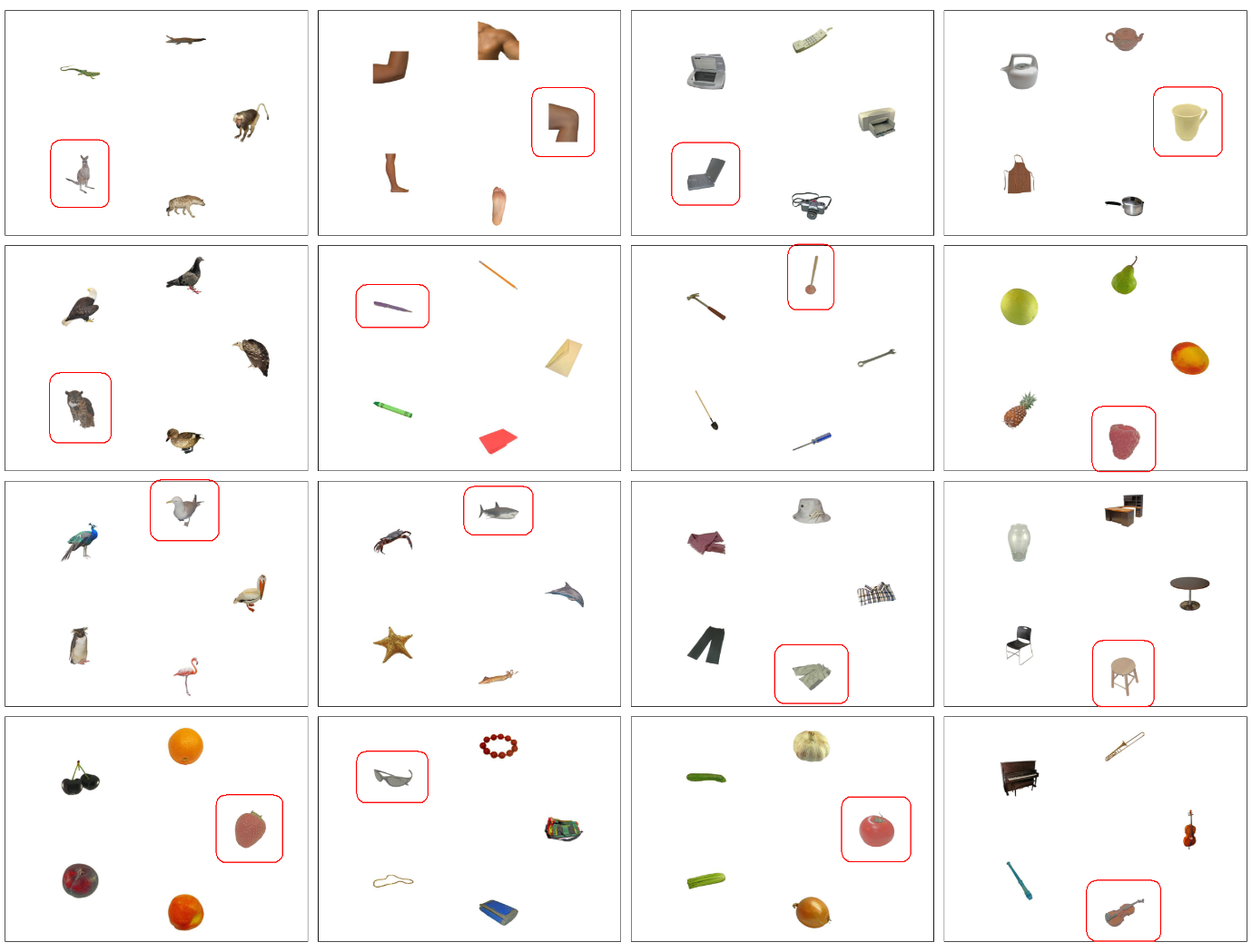


*Figure S12.* Experimental arrays with the critical objects (surrounded by their bounding boxes, in red) and distractors in the related and non-salient conditions, for set size 5 (32 arrays out of 32).

*Supplemental material continues*


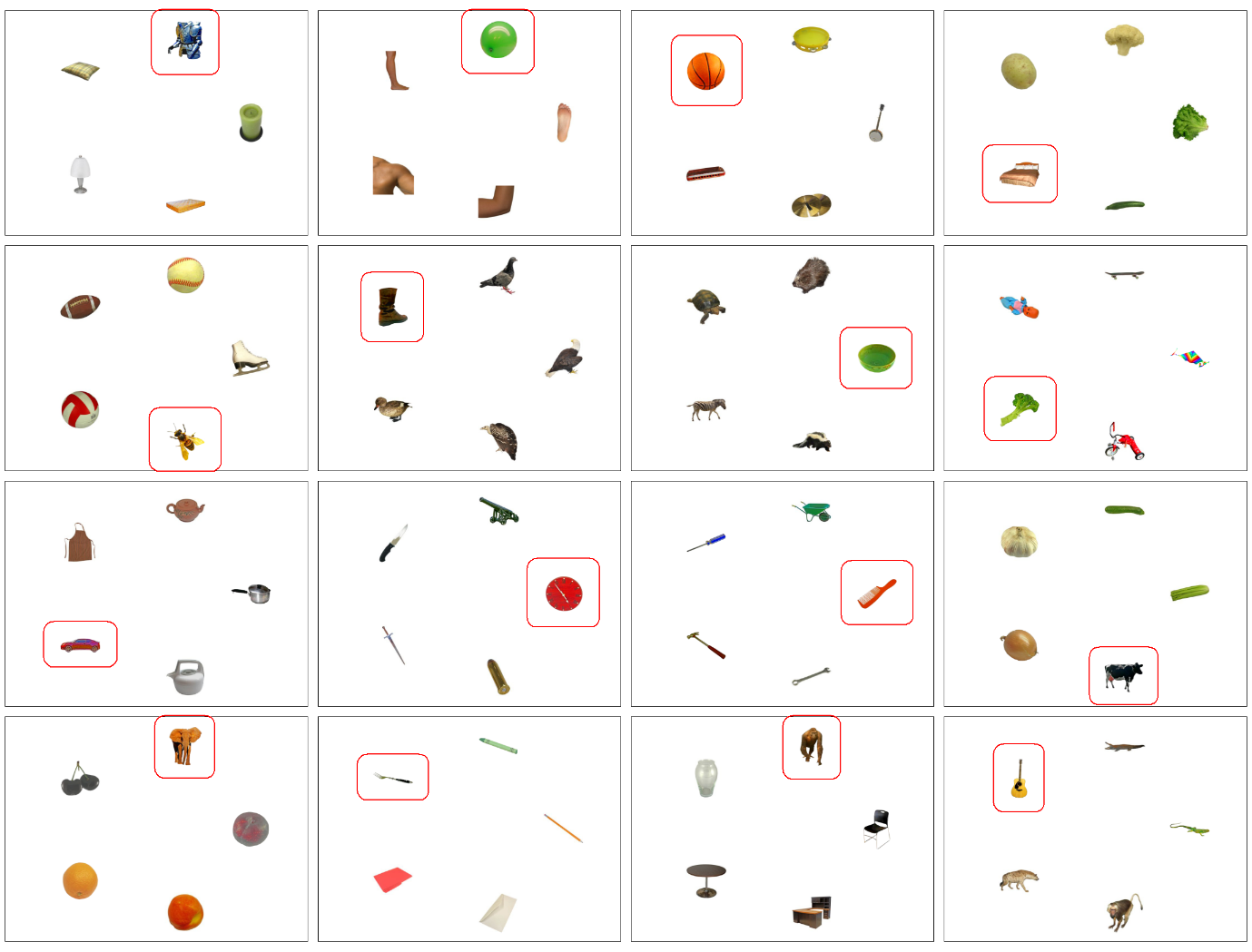


*Figure S13.* Experimental arrays with the critical objects (surrounded by their bounding boxes, in red) and distractors in the unrelated and salient conditions, for set size 5 (16 arrays out of 32).

*Supplemental material continues*


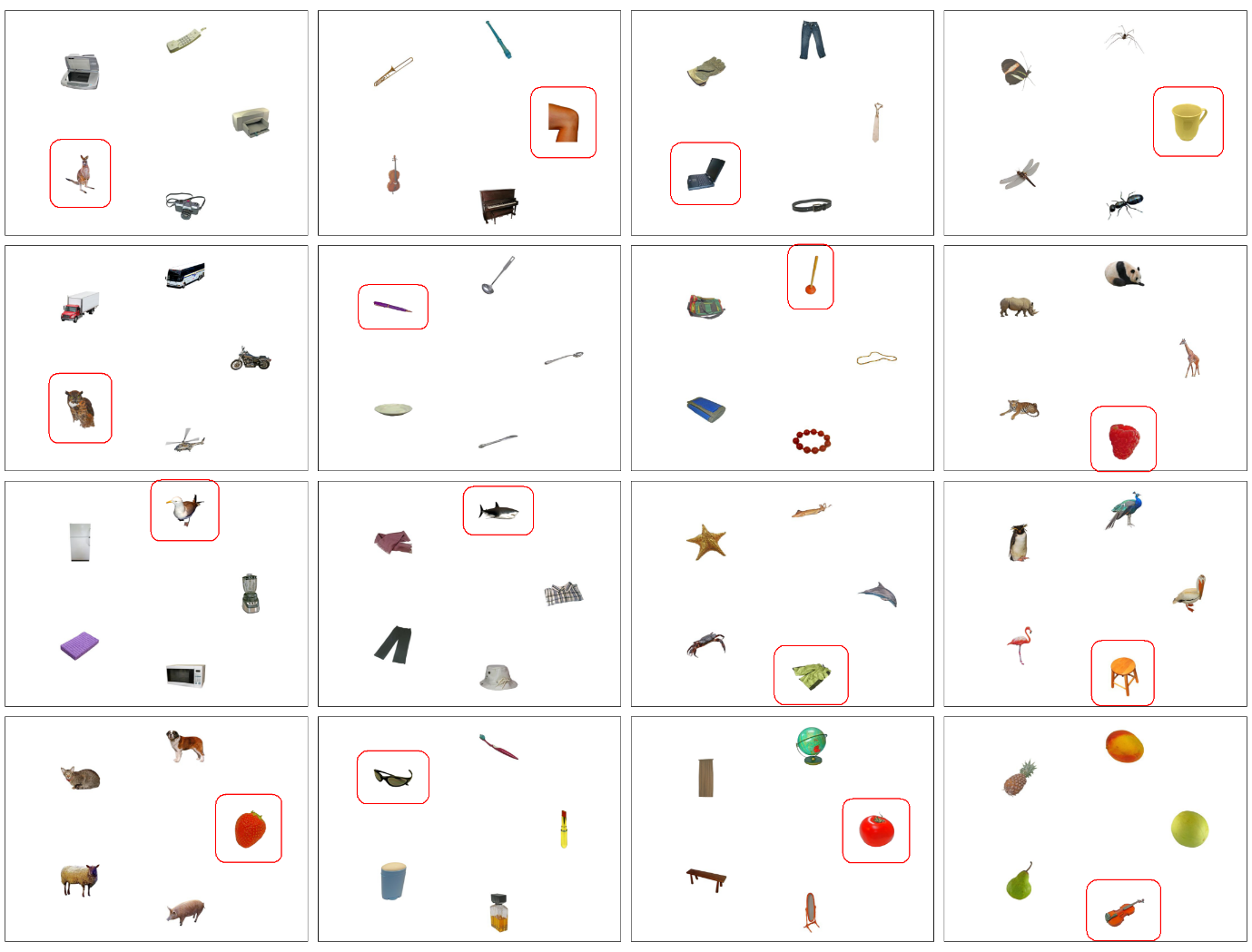


*Figure S14.* Experimental arrays with the critical objects (surrounded by their bounding boxes, in red) and distractors in the unrelated and salient conditions, for set size 5 (32 arrays out of 32).

*Supplemental material continues*


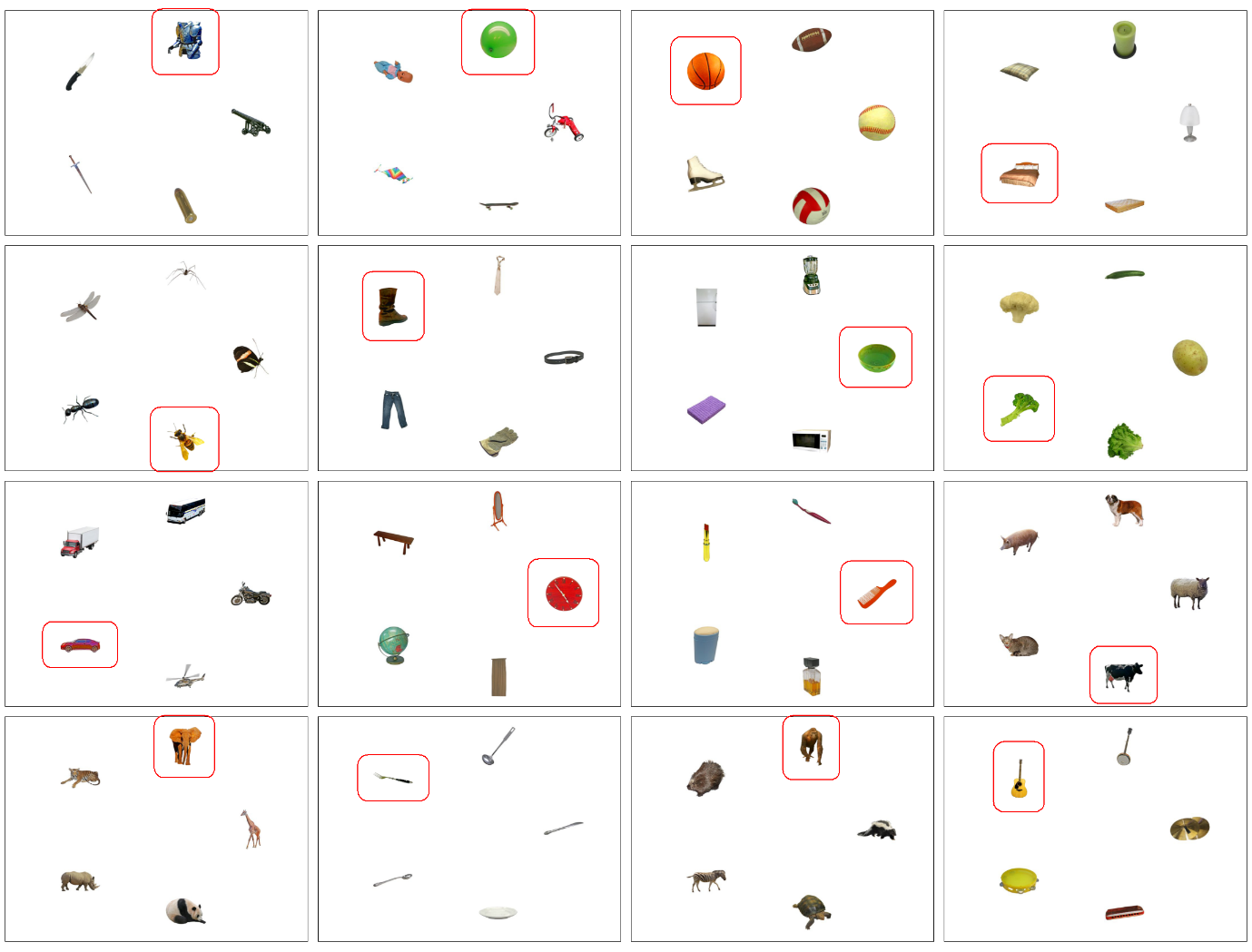


*Figure S15.* Experimental arrays with the critical objects (surrounded by their bounding boxes, in red) and distractors in the related and salient conditions, for set size 5 (16 arrays out of 32).

*Supplemental material continues*


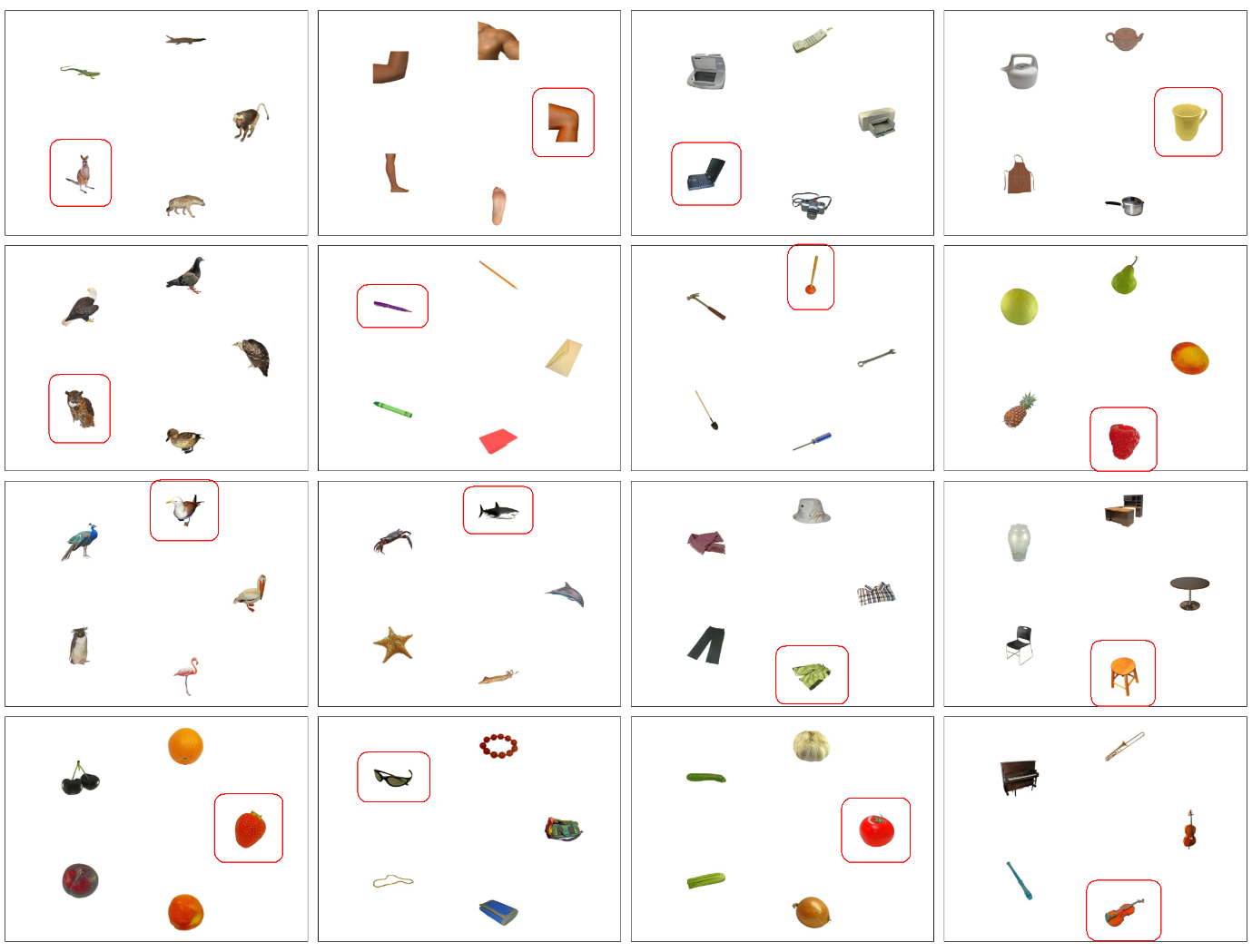


*Figure S16.* Experimental arrays with the critical objects (surrounded by their bounding boxes, in red) and distractors in the related and salient conditions, for set size 5 (32 arrays out of 32).

*Supplemental material continues*


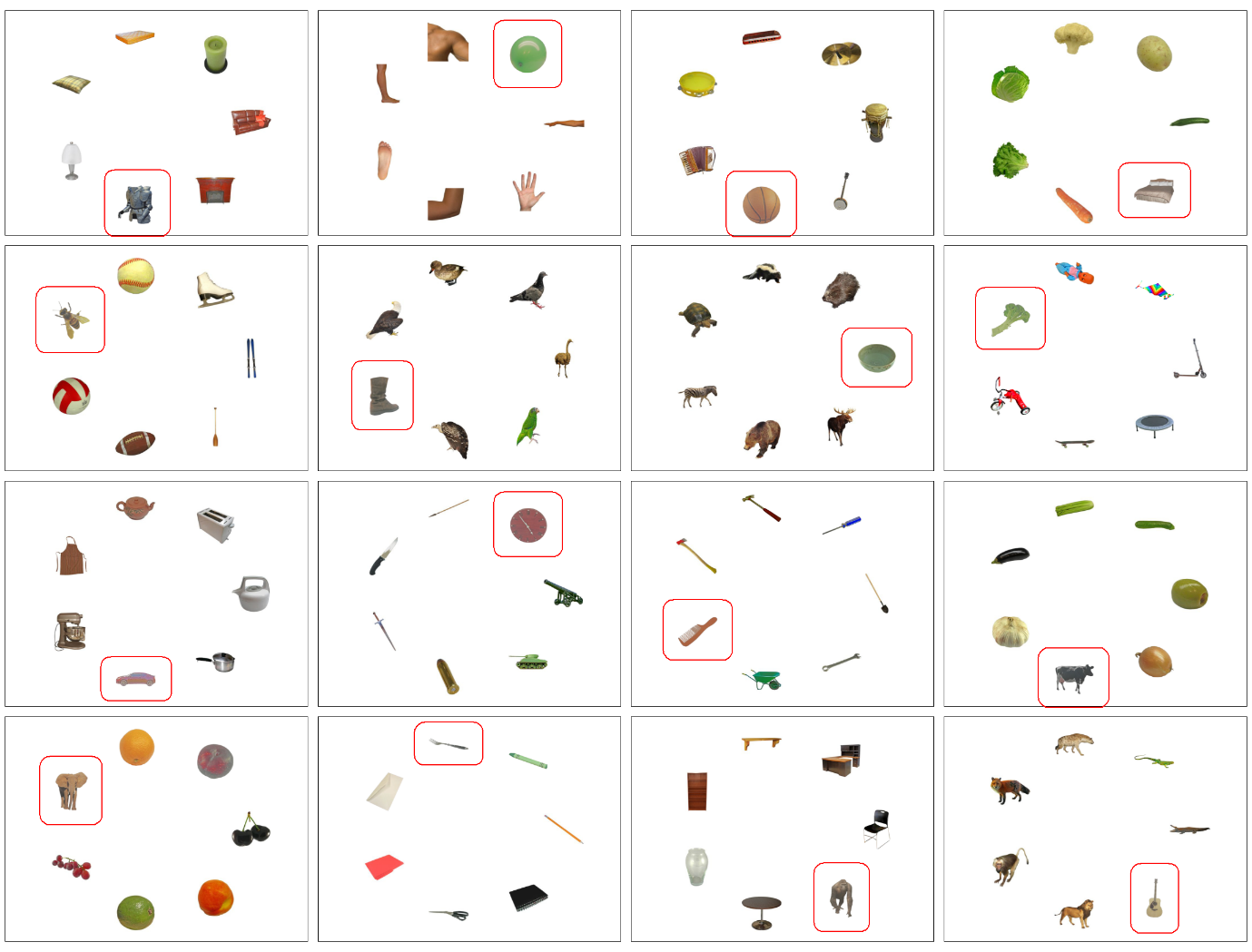


*Figure S17.* Experimental arrays with the critical objects (surrounded by their bounding boxes, in red) and distractors in the unrelated and non-salient conditions, for set size 7 (16 arrays out of 32).

*Supplemental material continues*


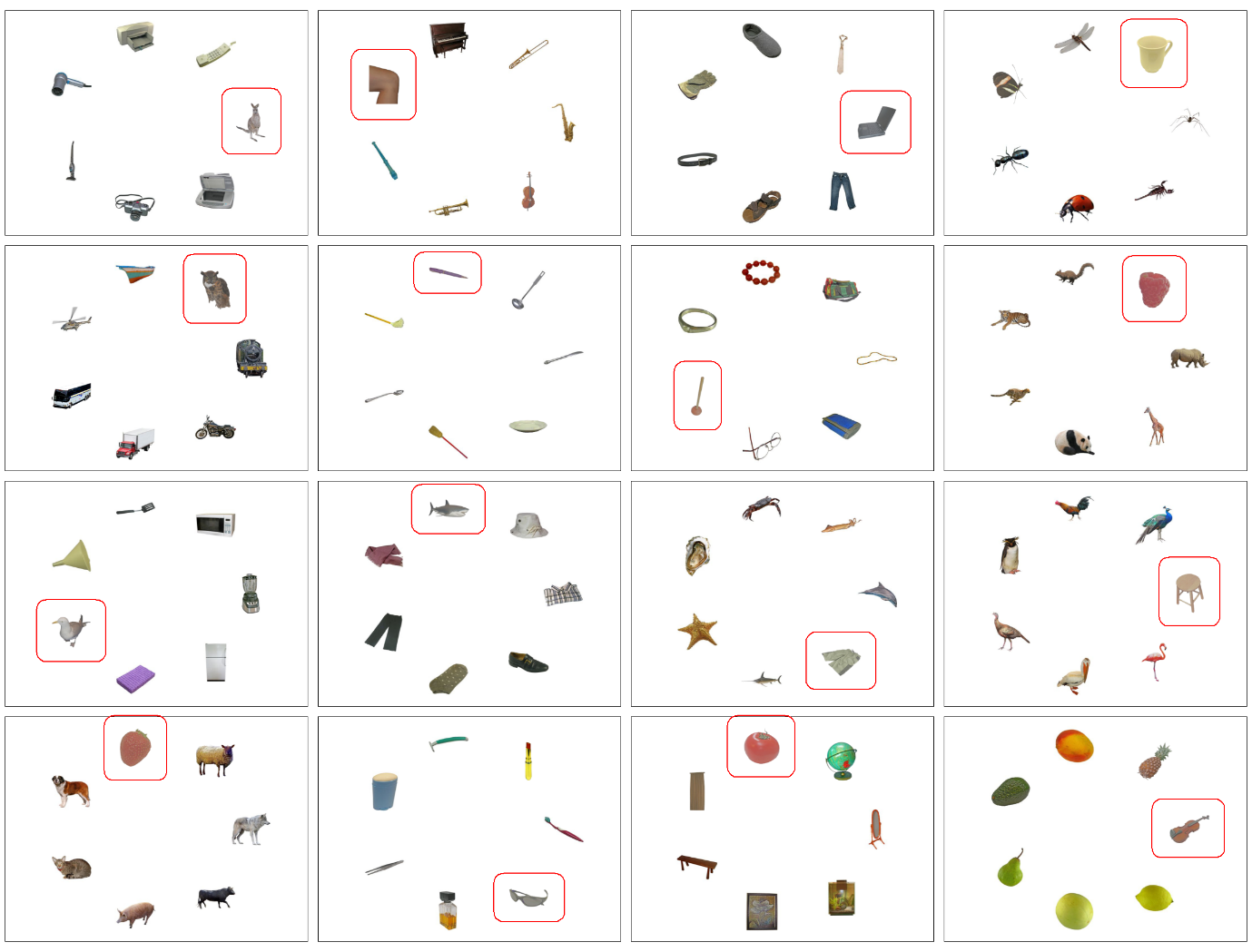


*Figure S18.* Experimental arrays with the critical objects (surrounded by their bounding boxes, in red) and distractors in the unrelated and non-salient conditions, for set size 7 (32 arrays out of 32).

*Supplemental material continues*


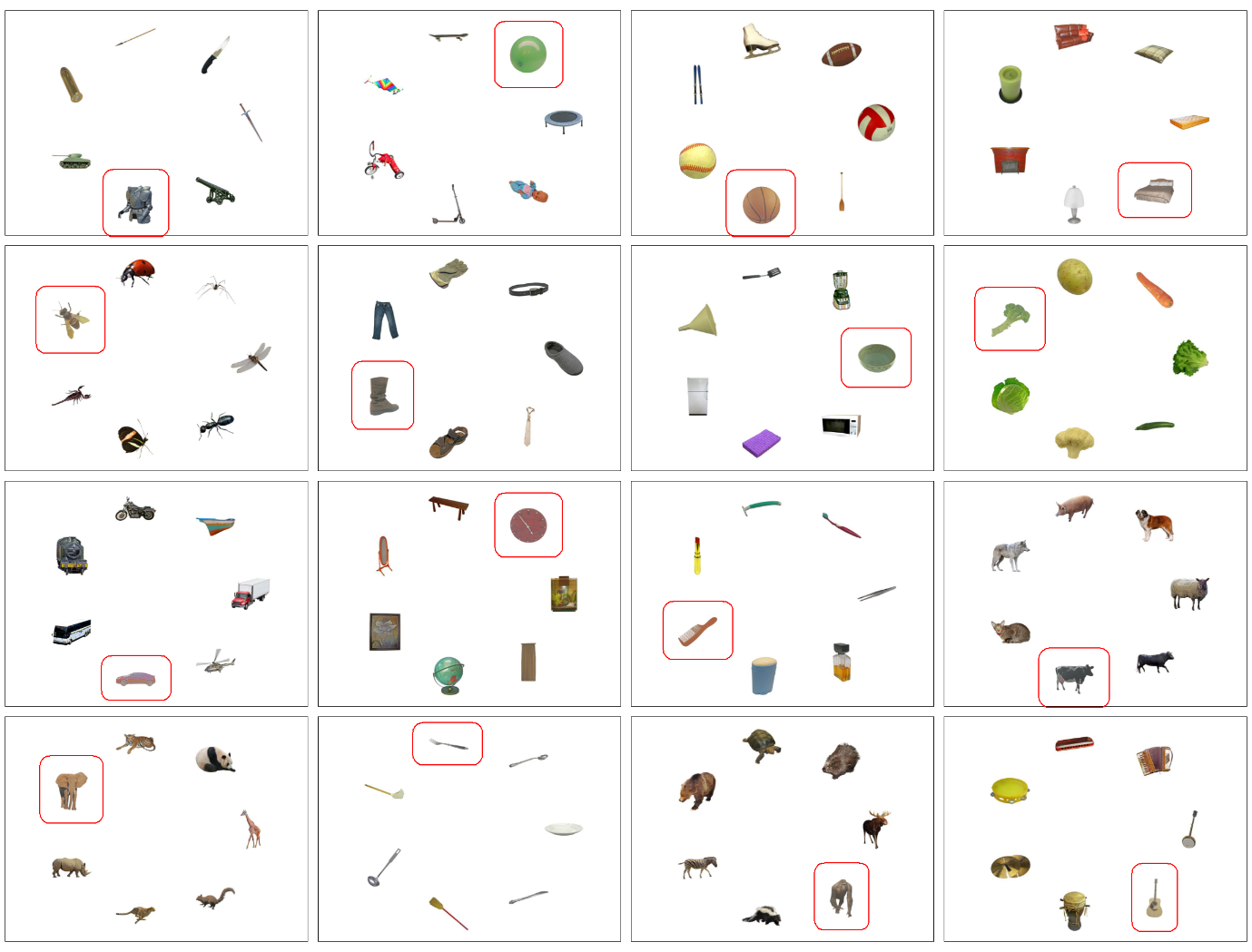


*Figure S19.* Experimental arrays with the critical objects (surrounded by their bounding boxes, in red) and distractors in the related and non-salient conditions, for set size 7 (16 arrays out of 32).

*Supplemental material continues*


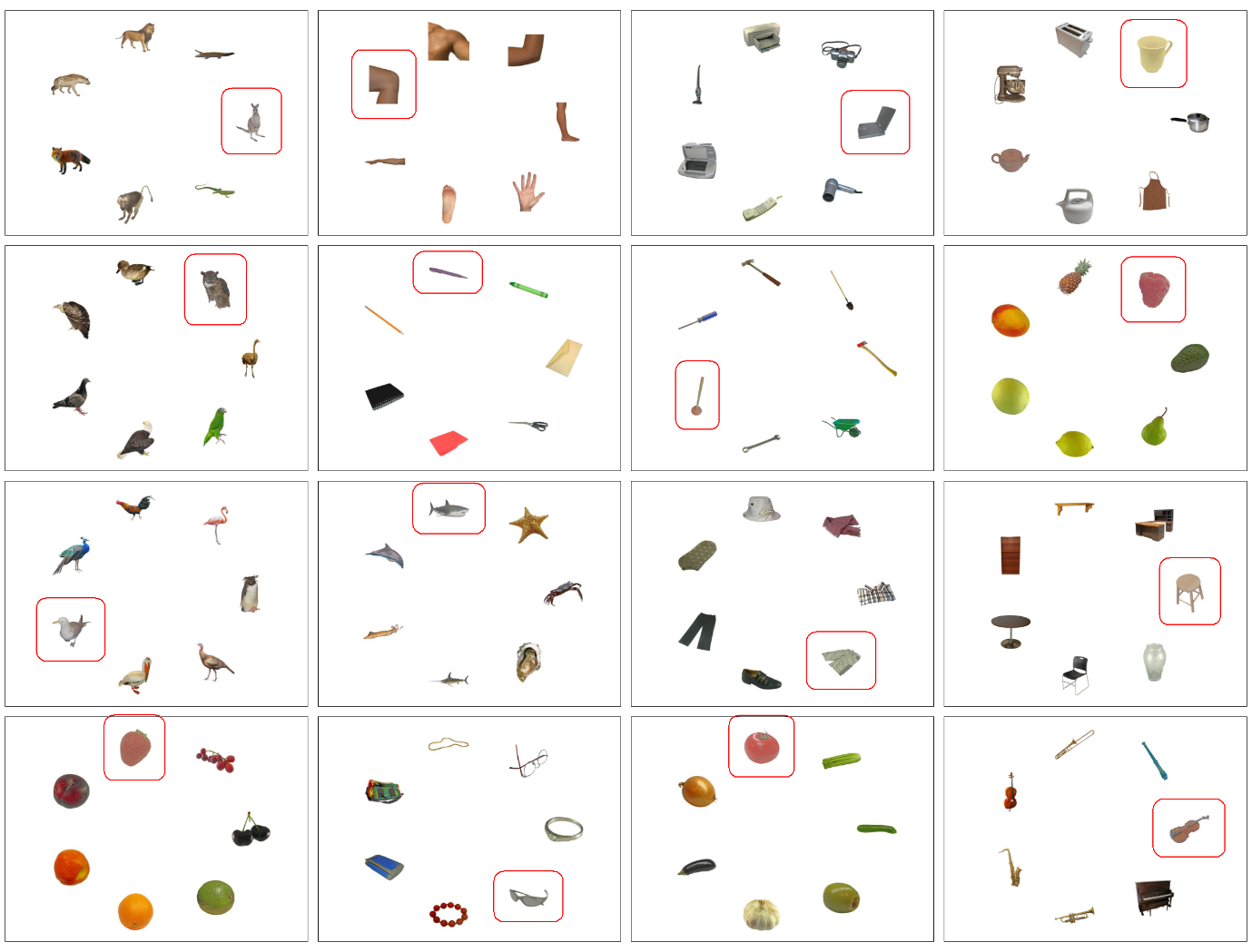


*Figure S20.* Experimental arrays with the critical objects (surrounded by their bounding boxes, in red) and distractors in the related and non-salient conditions, for set size 7 (32 arrays out of 32).

*Supplemental material continues*


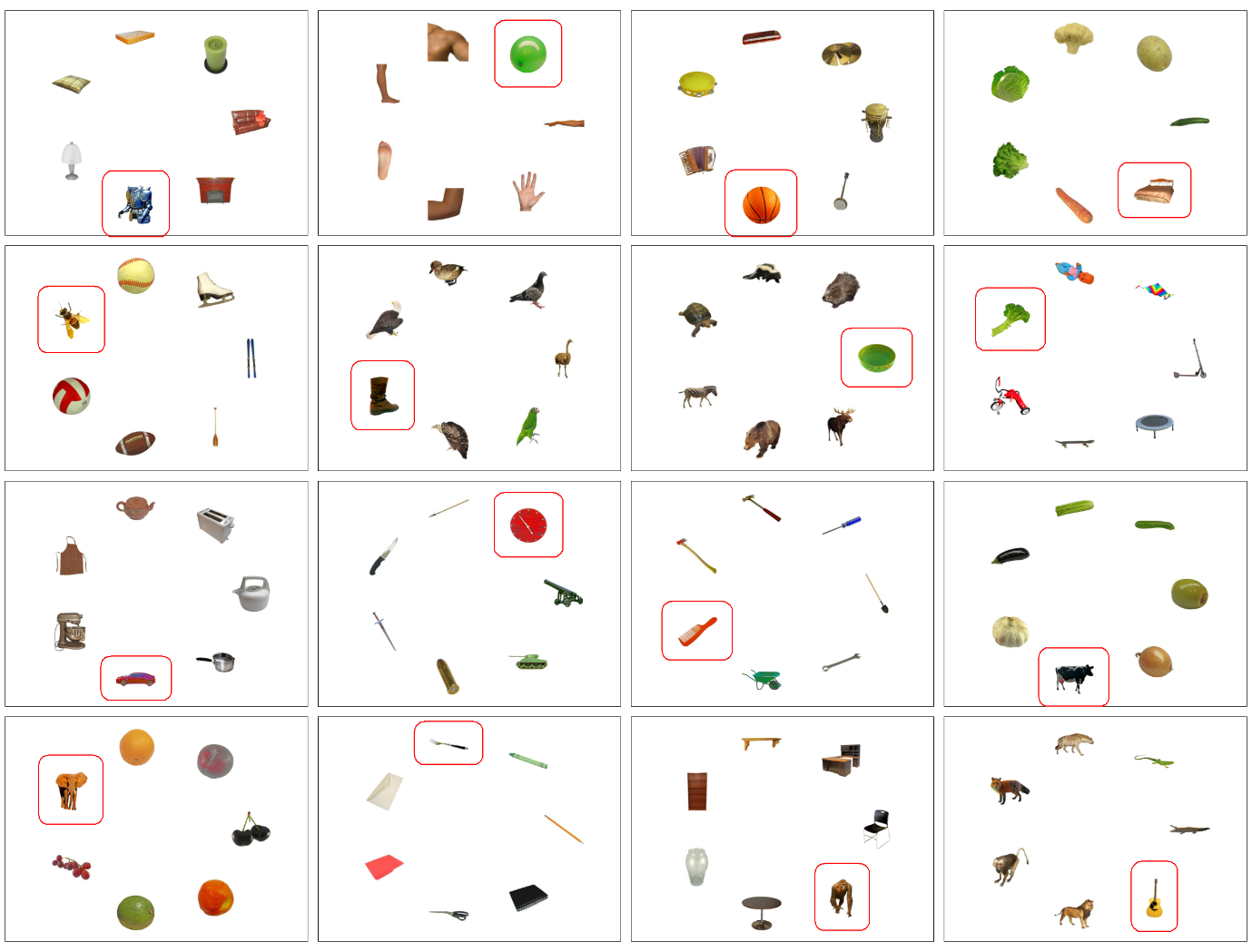


*Figure S21.* Experimental arrays with the critical objects (surrounded by their bounding boxes, in red) and distractors in the unrelated and salient conditions, for set size 7 (16 arrays out of 32).

*Supplemental material continues*


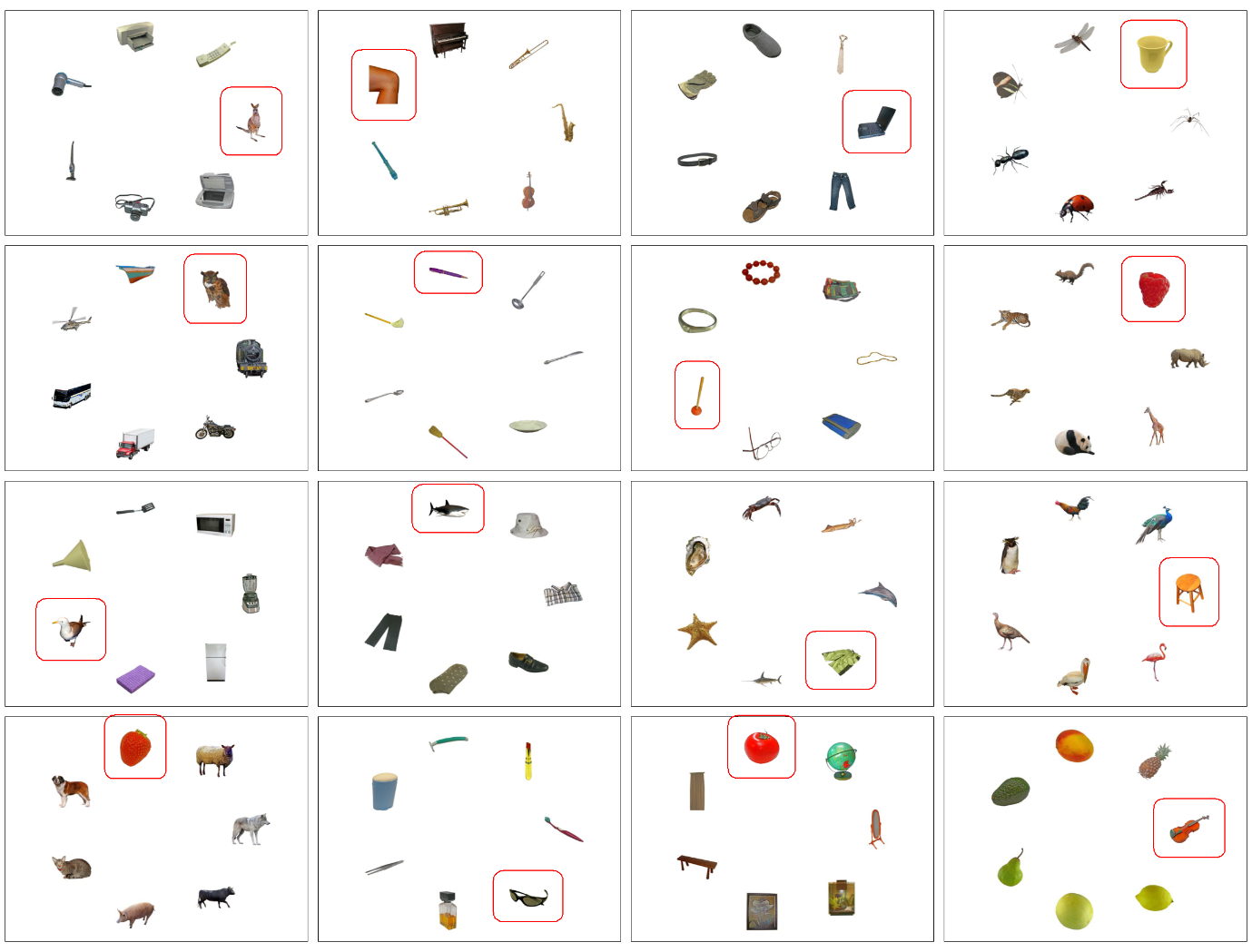


*Figure S22.* Experimental arrays with the critical objects (surrounded by their bounding boxes, in red) and distractors in the unrelated and salient conditions, for set size 7 (32 arrays out of 32).

*Supplemental material continues*


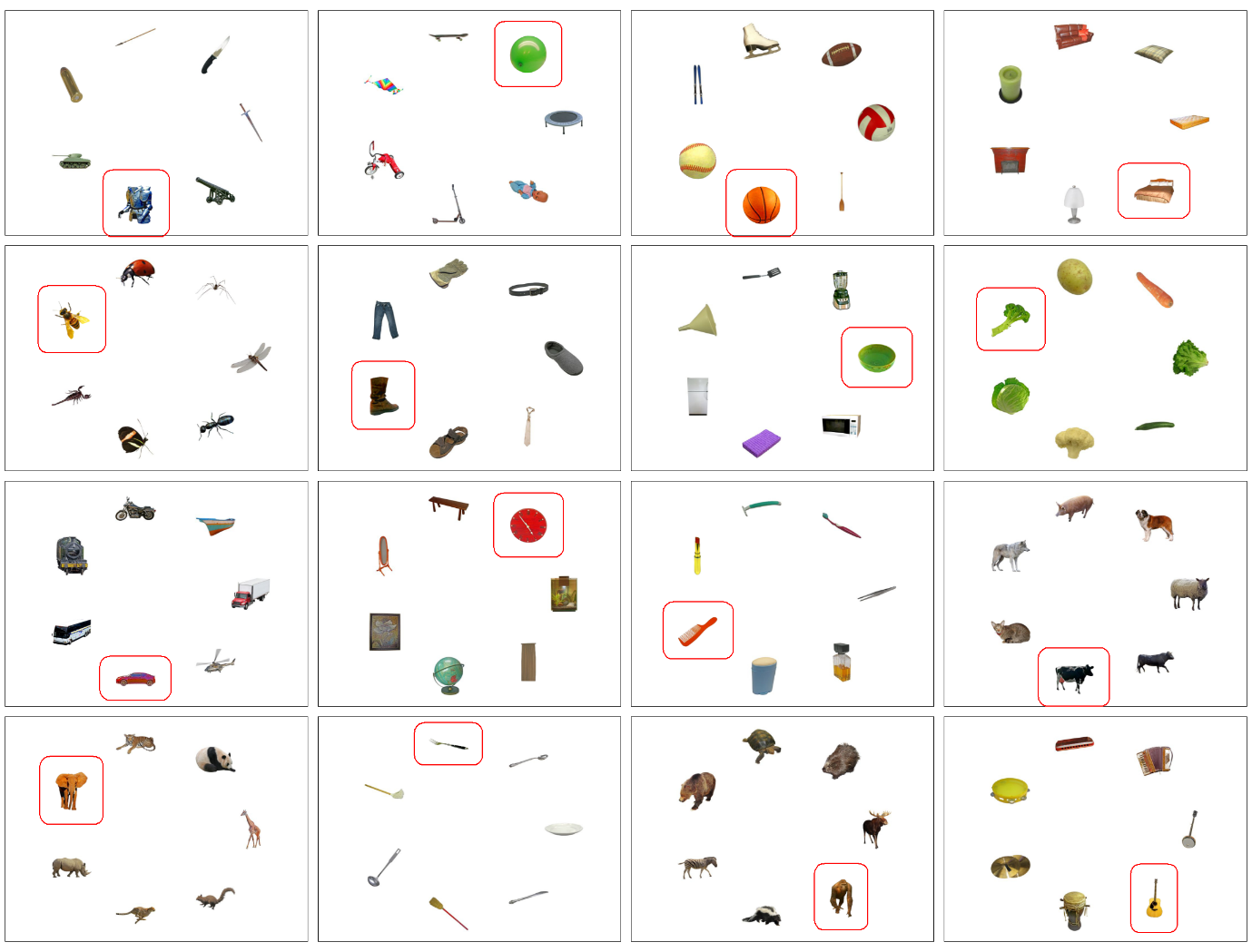


*Figure S23.* Experimental arrays with the critical objects (surrounded by their bounding boxes, in red) and distractors in the related and salient conditions, for set size 7 (16 arrays out of 32).

*Supplemental material continues*


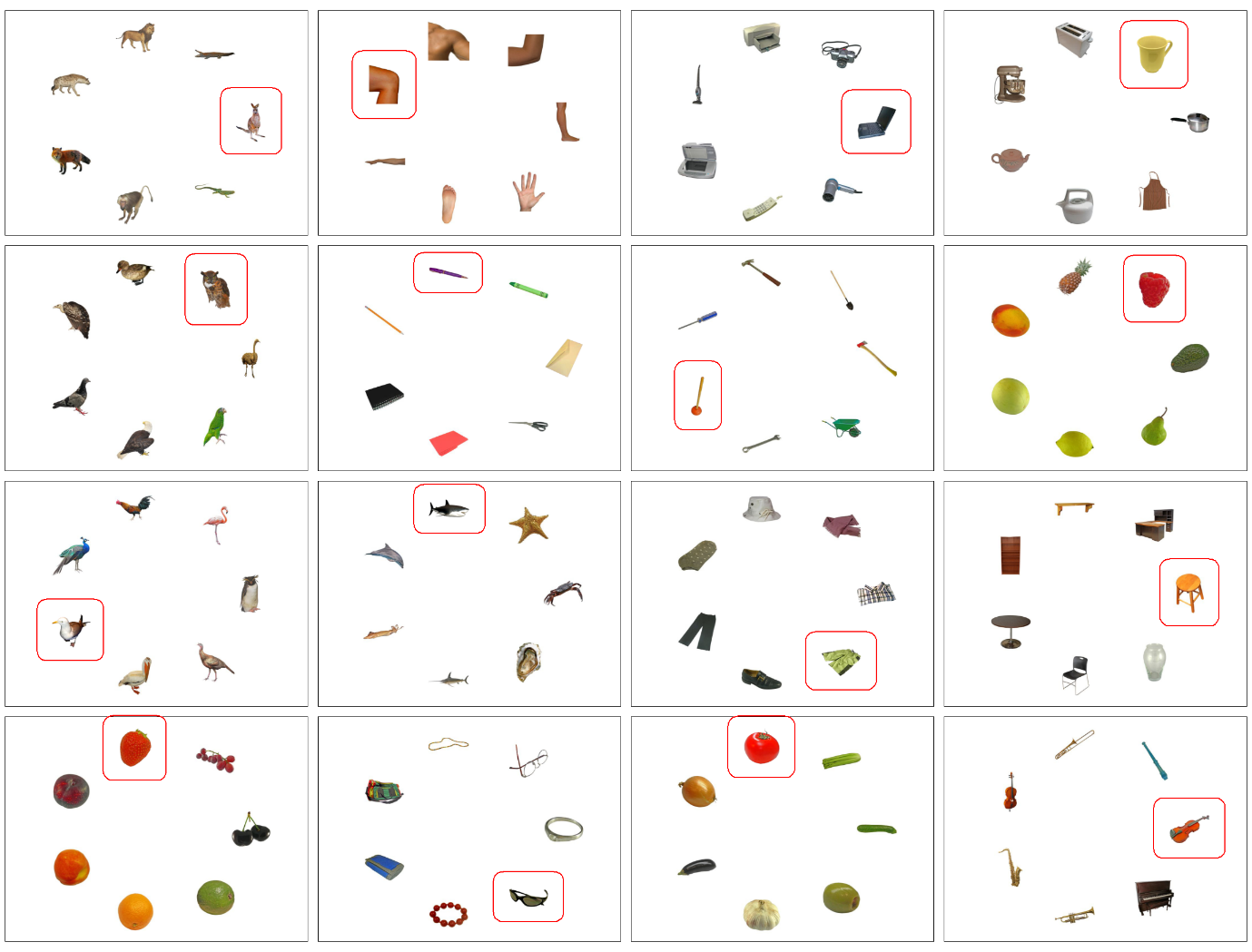


*Figure S24.* Experimental arrays with the critical objects (surrounded by their bounding boxes, in red) and distractors in the related and salient conditions, for set size 7 (32 arrays out of 32).

*Supplemental material continues*
