# Supplementary material for: Extra-foveal Processing of Object Semantics Guides Early Overt Attention During Visual Search
Source: Atten Percept Psychophys. 2019 Dec 2;82(2):655–70. doi: 10.3758/s13414-019-01906-1 (PMC7246246; doi:10.3758/s13414-019-01906-1)
Supplement: Supplementary file 3 — (DOCX 33.6 kb) [file 13414_2019_1906_MOESM3_ESM.docx]

**Supplemental Material C**

**List of Target-Present and -Absent Experimental Trials for Each Set Size by Semantic Relatedness Condition**

|  | Target name | |  | | Distractor object | | | |
| --- | --- | --- | --- | --- | --- | --- | --- | --- |
| Condition | Present-trials^a^ | Absent-  trials |  | |  | | | Set size 7 |
|  |  |  |  |  |  | Set size 5 | |  |
|  |  |  |  |  | Set size 3 | |  |  |
| Related | shark | prawn | | crab, squid | | | dolphin, starfish | oyster, swordfish |
| Unrelated |  |  | | shirt, trousers | | | hat, scarf | shoe, sock |
| Related | raspberry | banana | | pineapple, melon | | | pear, mango | lemon, avocado |
| Unrelated |  |  | | rhinoceros, tiger | | | giraffe, panda | cheetah, squirrel |
| Related | bowl | colander | | blender, microwave | | | fridge, sponge | spatula, funnel |
| Unrelated |  |  | | skunk, zebra | | | porcupine, turtle | bear, moose |
| Related | bed | dresser | | mattress, pillow | | | candle, lamp | couch, fireplace |
| Unrelated |  |  | | cauliflower, potato | | | cucumber, lettuce | cabbage, carrot |
| Related | basketball | treadmill | | baseball, volleyball | | | skate, football | paddle, skis |
| Unrelated |  |  | | harmonica, tambourine | | | banjo, cymbals | accordion, drum |
| Related | plunger | drill | | screwdriver, hammer | | | shovel, wrench | wheelbarrow, axe |
| Unrelated |  |  | | necklace, bracelet | | | bag, wallet | ring, glasses |
| Related | knee | nose | | elbow, leg | | | foot, shoulder | arm, hand |
| Unrelated |  |  | | cello, piano | | | flute, trombone | saxophone, trumpet |
| Related | tomato | mushroom | | celery, onion | | | courgette, garlic | aubergine, olive |
| Unrelated |  |  | | curtain, globe | | | bench, mirror | aquarium, painting |
| Related | violin | harp | | cello, piano | | | flute, trombone | saxophone, trumpet |
| Unrelated |  |  | | pineapple, melon | | | pear, mango | lemon, avocado |
| Related | owl | chicken | | eagle, vulture | | | duck, pigeon | ostrich, parrot |
| Unrelated |  |  | | truck, motorcycle | | | helicopter, bus | boat, train |

*Supplemental material continues*

|  | Target name | |  | Distractor object | | | | |
| --- | --- | --- | --- | --- | --- | --- | --- | --- |
| Condition | Present-trials | Absent-  trials |  |  | | | | Set size 7 |
|  |  |  |  |  | | Set size 5 | |  |
|  |  |  |  | Set size 3 | | |  |  |
| Related | sunglasses | watch | | | necklace, bracelet | | bag, wallet | ring, glasses |
| Unrelated |  |  | | | lipstick, perfume | | deodorant, toothbrush | razor, tweezers |
| Related | armour | arrow/  shield^b^ | | | cannon, bullet | | knife, sword | tank, spear |
| Unrelated |  |  | | | mattress, pillow | | candle, lamp | couch, fireplace |
| Related | comb | soap | | | lipstick, perfume | | deodorant, toothbrush | razor, tweezers |
| Unrelated |  |  | | | screwdriver, hammer | | shovel, wrench | wheelbarrow, axe |
| Related | bee | cockroach | | | butterfly, ant | | dragonfly, spider | ladybird, scorpion |
| Unrelated |  |  | | | baseball, volleyball | | skate, football | paddle, skis |
| Related | pen | calculator | | | crayon, pencil | | folder, envelope | notebook, scissors |
| Unrelated |  |  | | | plate, spoon | | knife, ladle | broom, mop |
| Related | balloon | swing | | | kite, tricycle | | doll, skateboard | trampoline, scooter |
| Unrelated |  |  | | | elbow, leg | | foot, shoulder | arm, hand |
| Related | laptop | television | | | printer, scanner | | camera, telephone | hoover, hairdryer |
| Unrelated |  |  | | | belt, jeans | | glove, tie | sandal, slipper |
| Related | shorts | coat | | | shirt, trousers | | hat, scarf | shoe, sock |
| Unrelated |  |  | | | crab, squid | | dolphin, starfish | oyster, swordfish |
| Related | car | airplane | | | truck, motorcycle | | helicopter, bus | boat, train |
| Unrelated |  |  | | | pot, teapot | | apron, kettle | mixer, toaster |
| Related | elephant | mouse | | | rhinoceros, tiger | | giraffe, panda | cheetah, squirrel |
| Unrelated |  |  | | | cherries, plum | | orange, peach | lime, grapes |
| Related | boot | jacket | | | belt, jeans | | glove, tie | sandal, slipper |
| Unrelated |  |  | | | eagle, vulture | | duck, pigeon | ostrich, parrot |

*Supplemental material continues*

|  | Target name | |  | Distractor object | | | | |
| --- | --- | --- | --- | --- | --- | --- | --- | --- |
| Condition | Present-trials | Absent-  trials |  |  | | | | Set size 7 |
|  |  |  |  |  | | Set size 5 | |  |
|  |  |  |  | Set size 3 | | |  |  |
| Related | mug | tray | | | pot, teapot | | apron, kettle | mixer, toaster |
| Unrelated |  |  | | | butterfly, ant | | dragonfly, spider | ladybird, scorpion |
| Related | stool | armchair | | | chair, table | | desk, vase | shelf, bookshelf |
| Unrelated |  |  | | | flamingo, penguin | | peacock, pelican | rooster, turkey |
| Related | broccoli | pumpkin | | | cauliflower, potato | | cucumber, lettuce | cabbage, carrot |
| Unrelated |  |  | | | kite, tricycle | | doll, skateboard | trampoline, scooter |
| Related | guitar | clarinet | | | harmonica, tambourine | | banjo, cymbals | accordion, drum |
| Unrelated |  |  | | | lizard, crocodile | | hyena, monkey | fox, lion |
| Related | seagull | swan | | | flamingo, penguin | | peacock, pelican | rooster, turkey |
| Unrelated |  |  | | | blender, microwave | | fridge, sponge | spatula, funnel |
| Related | strawberry | apple | | | cherries, plum | | orange, peach | lime, grapes |
| Unrelated |  |  | | | pig, sheep | | cat, dog | bull, wolf |
| Related | cow | horse | | | pig, sheep | | cat, dog | bull, wolf |
| Unrelated |  |  | | | celery, onion | | courgette, garlic | aubergine, olive |
| Related | kangaroo | rabbit | | | lizard, crocodile | | hyena, monkey | fox, lion |
| Unrelated |  |  | | | printer, scanner | | camera, telephone | hoover, hairdryer |
| Related | fork | pan | | | plate, spoon | | knife, ladle | broom, mop |
| Unrelated |  |  | | | crayon, pencil | | folder, envelope | notebook, scissors |
| Related | clock | rug | | | curtain, globe | | bench, mirror | aquarium, painting |
| Unrelated |  |  | | | cannon, bullet | | knife, sword | tank, spear |
| Related | gorilla | hippopotamus | | | skunk, zebra | | porcupine, turtle | bear, moose |
| Unrelated |  |  | | | chair, table | | desk, vase | shelf, bookshelf |

*Note.*

^a^The 32 critical objects are listed in the target name column for target-present trials.

^b^Shield replaced arrow as target name for target-absent trials in set size 7.
